# Evaluating transurethral resection of the prostate over twenty years: a systematic review and meta-analysis of randomized clinical trials

**DOI:** 10.1007/s00345-024-05332-3

**Published:** 2024-11-15

**Authors:** Joao G. Porto, Ansh M. Bhatia, Abhishek Bhat, Maria Camila Suarez Arbelaez, Ruben Blachman-Braun, Khushi Shah, Ankur Malpani, Diana Lopategui, Thomas R. W. Herrmann, Robert Marcovich, Hemendra N. Shah

**Affiliations:** 1 Desai Sethi Urology Institute, University of Miami, Miller School of Medicine, Miami, USA; 2 Spitial Thurgau AG (STGAG), Frauenfeld, Thurgau Switzerland

**Keywords:** Transurethral resection of the prostate, Benign prostatic hyperplasia, Enlarged prostate, Lower urinary tract symptoms

## Abstract

**Purpose:**

The goal of this systematic review is to assess the temporal changes in outcomes and complications of transurethral resection of the prostate (TURP) from 2000 to 2022.

**Methods:**

We conducted a systematic review and meta-analysis of 103 randomized clinical trials from PubMed on TURP, involving 8521 patients. Studies were grouped by years: 2000–2004, 2005–2009, 2010–2014, and 2015–2022. We assessed International Prostate Symptom Score (IPSS), Peak Flow (Qmax), Post-void residue of urine (PVR), and post-operative complications. Heterogeneity was ranked as low (I^2^ < 25%), moderate (I^2^ = 25–75%), or high (I^2^ > 75%).

**Results:**

TURP significantly improved IPSS, Qmax, and PVR, with the most recent studies showing superior results in IPSS and Qmax after 3 years compared to 2000–2004 studies. Heterogeneity in PVR was high (I^2^ = 100%). No negative impact on erectile function was observed. Complication rates included TURP syndrome (2%), bleeding (8%), and blood transfusion (6%), but elevated heterogeneity with difference between the groups was seen in clot evacuation (I^2^ = 83%) and urinary tract infections (I^2^ = 82%). Other complications were urinary retention (4%), incontinence (8%), urethral stricture (3%), bladder neck stenosis (2%).

**Conclusion:**

In the last 20 years there has not been a clear trend in the results of TURP. The found heterogeneity may indicate a lack of standardization in TURP procedures. However, symptomatic improvement among patients is uniform, which supports this procedure as a historical benchmark surgical treatment for BPH.

**Supplementary Information:**

The online version contains supplementary material available at 10.1007/s00345-024-05332-3.

## Introduction

Benign prostatic hyperplasia (BPH) is a leading cause of bladder outlet obstruction (BOO) in men, with transurethral resection of the prostate (TURP) historically regarded as the gold standard for surgical management [1]. Although minimally invasive surgical therapies (MIST) have emerged, offering fewer complications, and enucleation procedures have advanced, demonstrating lower reoperation rates and better functional outcomes, TURP remains widely practiced due to its proven efficacy, easy availability, cost effectivity, and important part of urological training [2–5]. However, TURP has notable limitations, including its size dependency and unsuitability for patients on blood thinners.

In the last two decades, TURP has undergone significant technological advancements, including improvements in endoscopic equipment and surgical techniques. While some studies have shown that these advancements have reduced complications, findings have been inconsistent, potentially due to variations in surgeon experience [6–9].

Due to the emergence of new techniques and variations in trends regarding TURP worldwide, we hypothesized that the quality of care provided by TURP practitioners, and the likelihood of complications significantly differ across periods. To address these concerns, we conducted a systematic review and meta-analysis of randomized clinical trials (RCT) with studies conducted in the twenty-first century.

## Aims

Our objective was to comprehensively evaluate the efficacy and safety of this well-established treatment and identify any potential patterns to assess the best practices related to TURP.

## Evidence acquisition

### Search strategy

A PubMed search was conducted in March 2023 with the keywords: “transurethral resection of the prostate”, “benign prostatic hyperplasia”, “enlarged prostate”, and “randomized clinical trial”. We searched the PubMed database from January 1st, 2000, until December 31st, 2022. Only RCTs published in English language which compared TURP to other treatments were included. We excluded systematic review articles, meta-analyses and duplicated articles, case reports, expert opinion articles, and commentaries. All studies were included regardless of prostate size.

### Data extraction

We collected data related to International Prostate Symptom Score (IPSS), maximum urine flow rate (Qmax), postvoid residual volume (PVR), prostate-specific-antigen (PSA), prostate volume, Sexual Health Inventory For Men (SHIM), and perioperative complications; such as transurethral resection syndrome, bleeding, blood transfusion, clot evacuation, urinary retention, urinary tract infection (UTI), irritative symptoms, urinary incontinence, erectile dysfunction (ED), retrograde ejaculation, urethral stricture (US), and bladder neck stenosis (BNS). We also evaluated the rate of incidental prostate cancer (iPCa) and retreatment at 1 and 3 years after TURP. Perioperative outcomes were recorded and represented in a tabular format. Data was grouped in time points at baseline, 3-months, 1-year, and > 3-years.

### Data analysis and synthesis

The selected articles were divided into four groups according to year of publication for analysis: 2000–2004, 2005–2009, 2010–2014, and 2015–2022. This division aimed to provide a comprehensive overview of TURP outcomes throughout the twenty-first century, highlighting differences every five years. However, to make study more up to date we extended the last group to more than five years.

The mean difference (MD) for change from baseline at each timepoint was calculated as an outcome measure for continuous variables and compared across all studies and between the groups. The I^2^ statistic was used to assess heterogeneity, with < 25% indicating low heterogeneity, 25–75% indicating moderate heterogeneity, and > 75% indicating high heterogeneity. The Qm statistic was used to quantify group heterogeneity, and a *p*-value < 0.05 was considered significant. Tau^2^ and Cochrane’s Q were also calculated for groups and studies. These were reported as forest plots. Proportions of complications with 95% confidence intervals were calculated, and heterogeneity was assessed for each complication separately when stratified by time groups. The pooled proportion estimate was calculated using a random effect model. Forest plots were used to visualize the outcome. This analysis was performed In RStudio (RStudio Inc, MA, USA), using the “metafor” package.

## Results and discussion

### Study selection

Selected articles were reviewed with full-text, and 103 studies were included and deemed eligible for review [9–111]. All disagreements between authors were settled by discussion and consensus. Two authors (AB, MCS) independently reviewed the articles’ titles and abstracts. The data was extracted by AB and MCS independently and cross-verified. For each article included the year of publication was considered year of data collection. This study is registered with the PROSPERO database (CRD42023401743) and was conducted following the Preferred Reporting Items for Systematic Reviews and Meta-analyses (PRISMA) guidelines. The present study offers a temporal analysis of this registration. Supplementary Fig. 1 shows the PRISMA diagram.

### Selected studies

We found 103 studies with 8521 patients that originated from 2000 to 2004 (n = 17), 2005–2009 (n = 17), 2010–2014 (n = 37), and 2015–2022 (n = 33). The comparator for TURP in these studies is shown in Supplementary Table 1. There were more studies comparing TURP with minimally invasive techniques in later half of study period.

### Voiding outcomes: IPSS, Qmax, and PVR

**Fig. 1:**
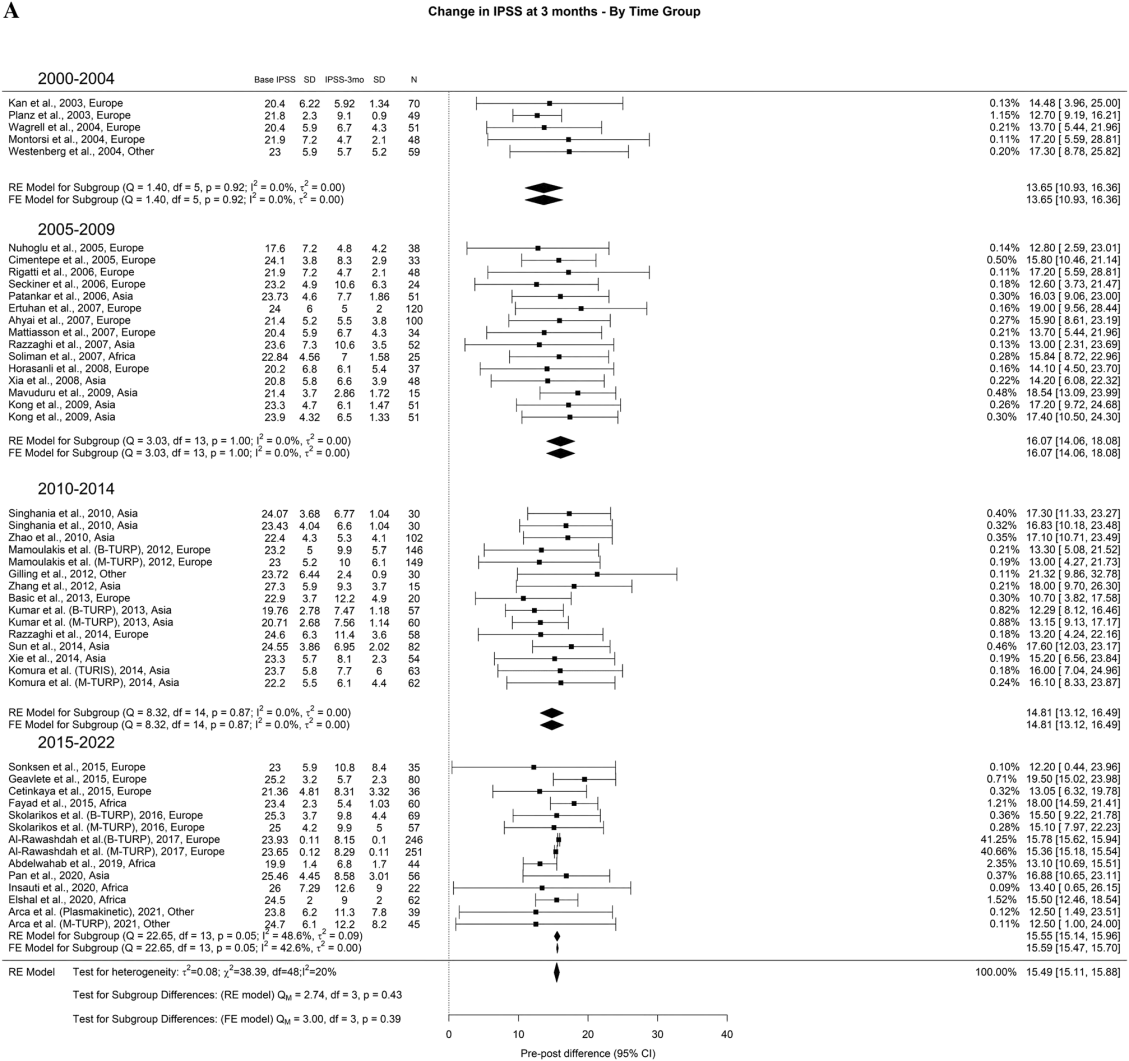

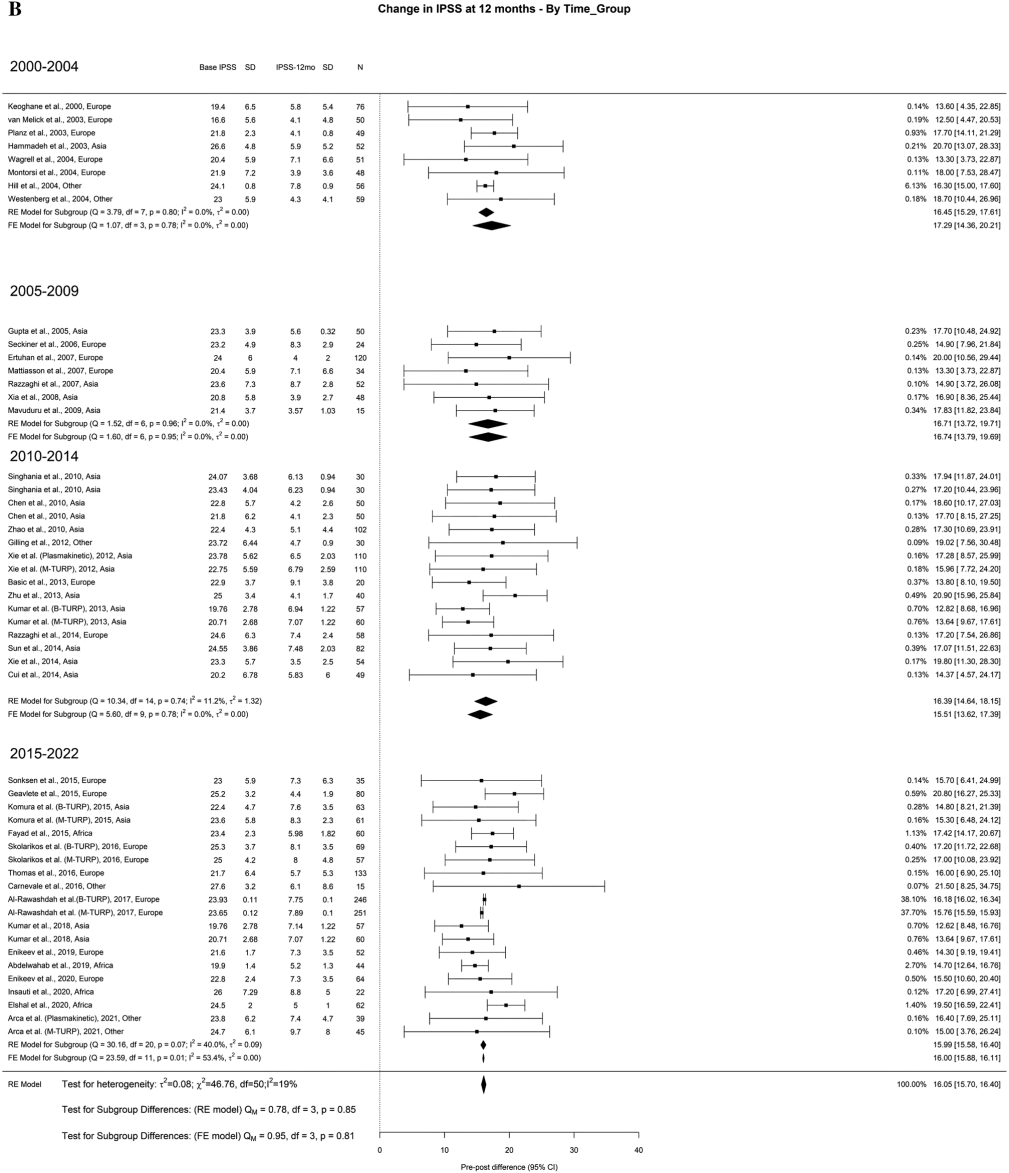

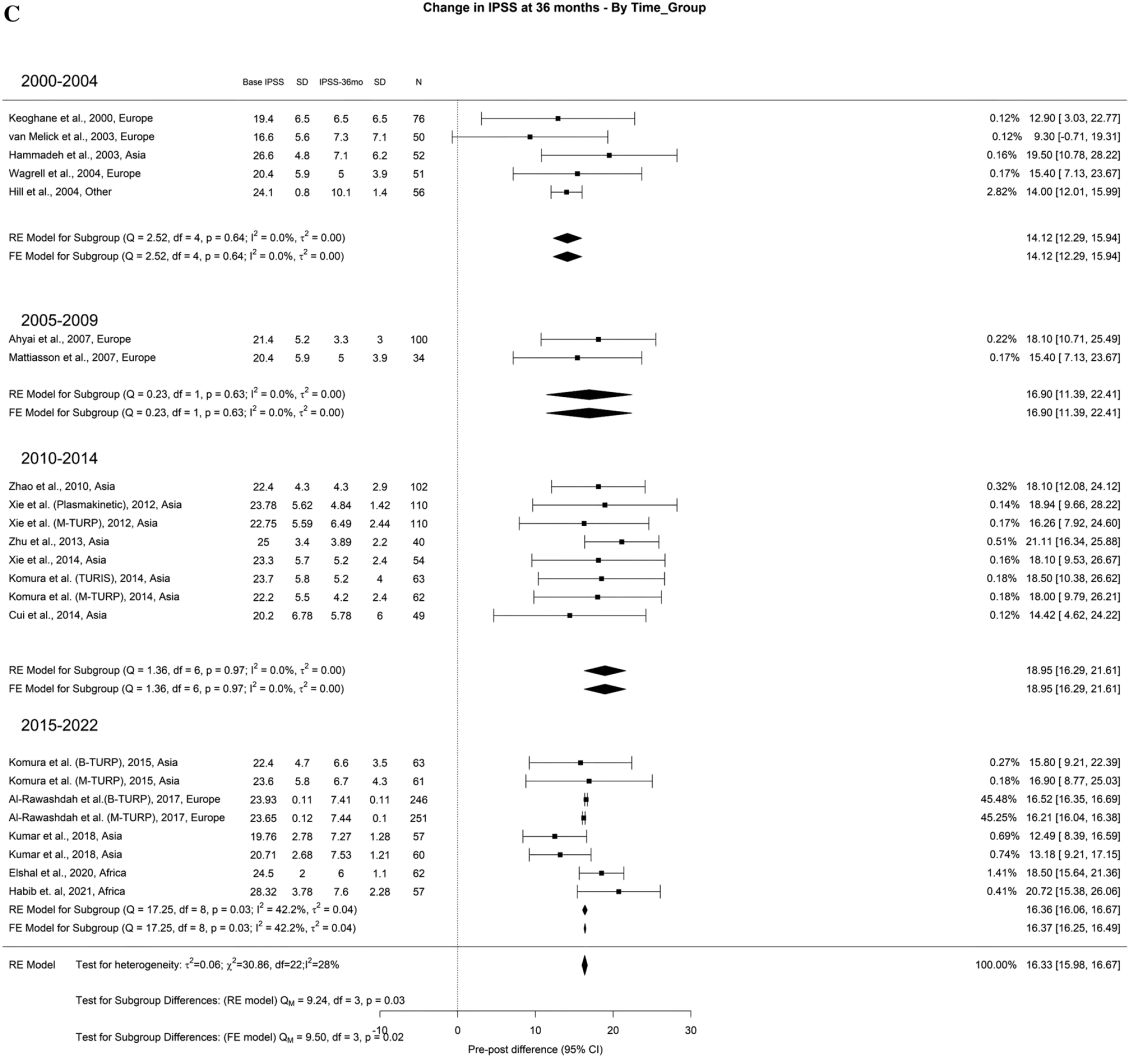
Change in the IPSS by time group. A. At 3 months; B. At 12 months; C. At 36 months

The overall average decrease in IPSS at 3-month, 1-year, and > 3-years was 15.4 points, 16 points, and 16.3 points, respectively. We found that > 3-years, TURP had better results in the most recent timeframes when compared to 2000–2004 studies. The drop in IPSS at > 3 years for studies from 2000–2004 was 14.12 points, while the decreases in 2005–2009, 2010–2014, and 2015–2022 were 16.90 points, 18.95 points, and 16.36 points, respectively (Fig. 1A–C).

**Fig. 2:**
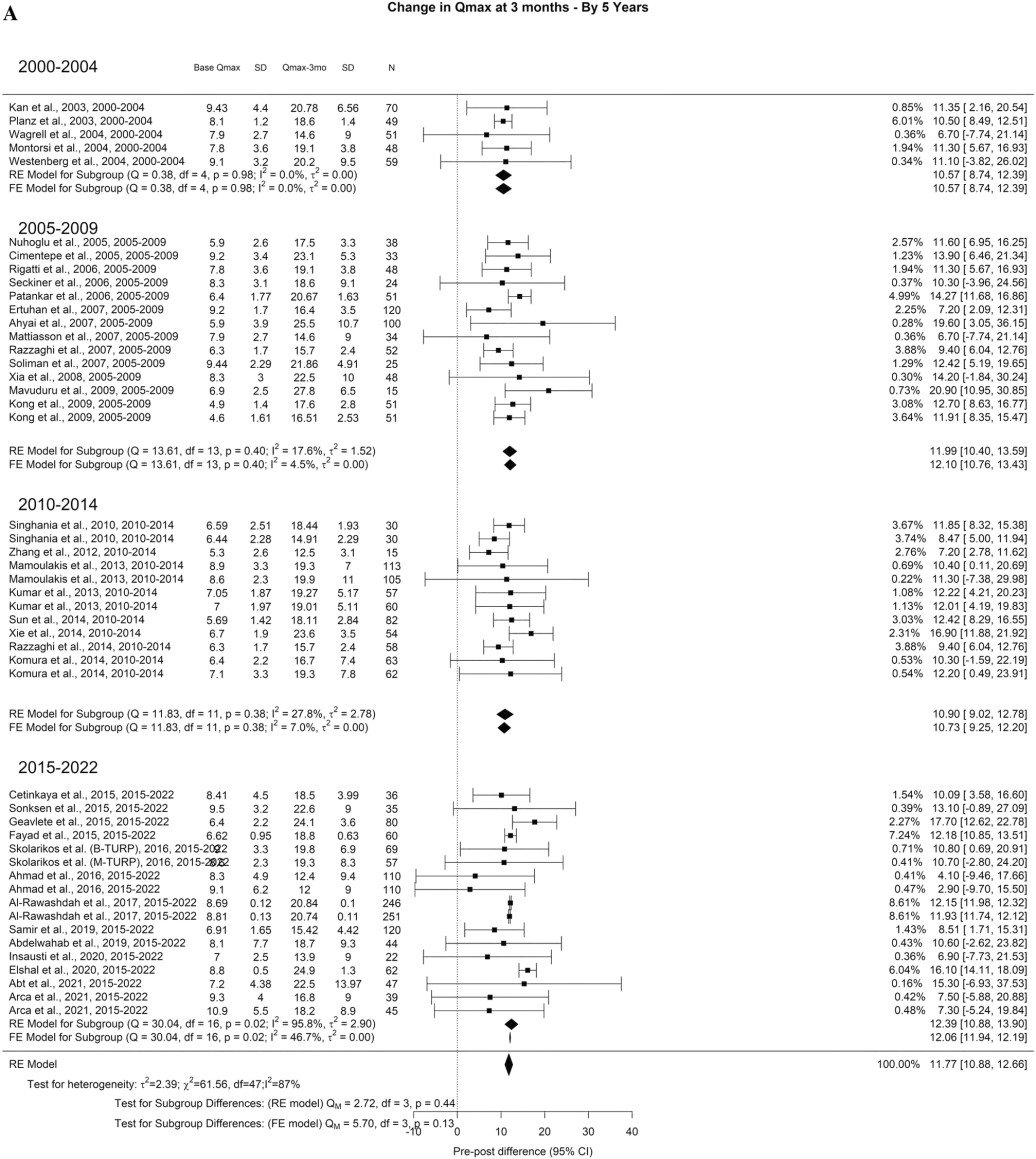

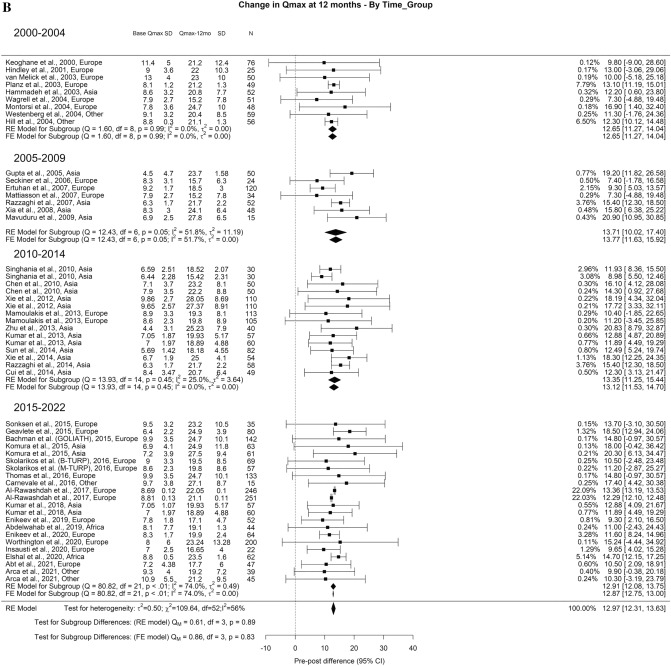

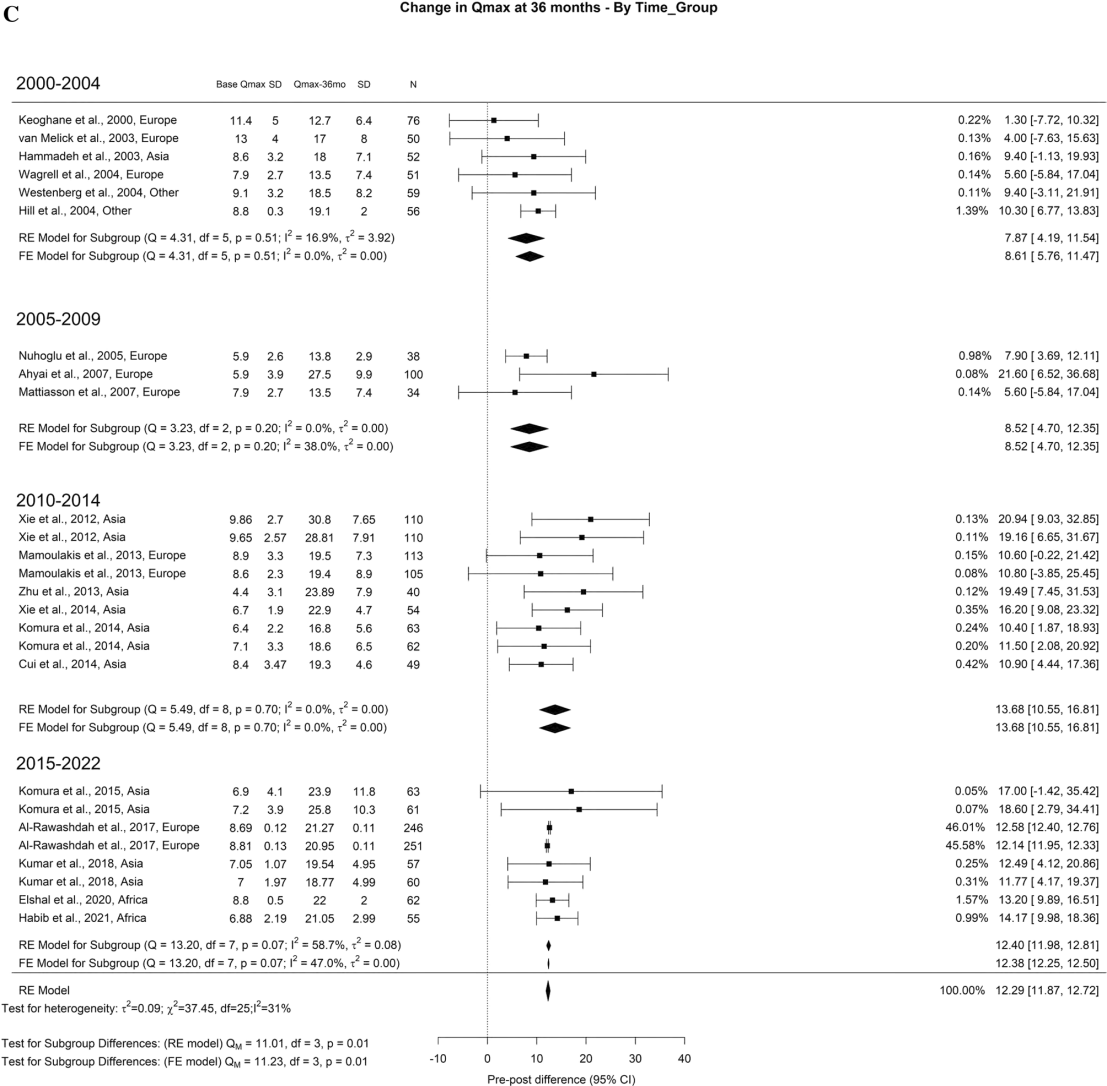
Change in the Qmax by time group. A. At 3 months; B. At 12 months; C. At 36 months

Related to Qmax, the overall improvement at 3-month, 1-year, and > 3-year was 11.77 ml/s, 12.97 ml/s, 12.29 ml/s, respectively. A significant difference was seen at > 3-years (Qm = 11.1, *p* = 0.01). The Qmax improvement at > 3-years was: 7.87 ml/s for 2000–2004, 8.52 ml/s for 2005–2009, 13.68 ml/s for 2010–2014, and 12.40 ml/s for 2015–2022 (Fig. 2A–C).

**Fig. 3:**
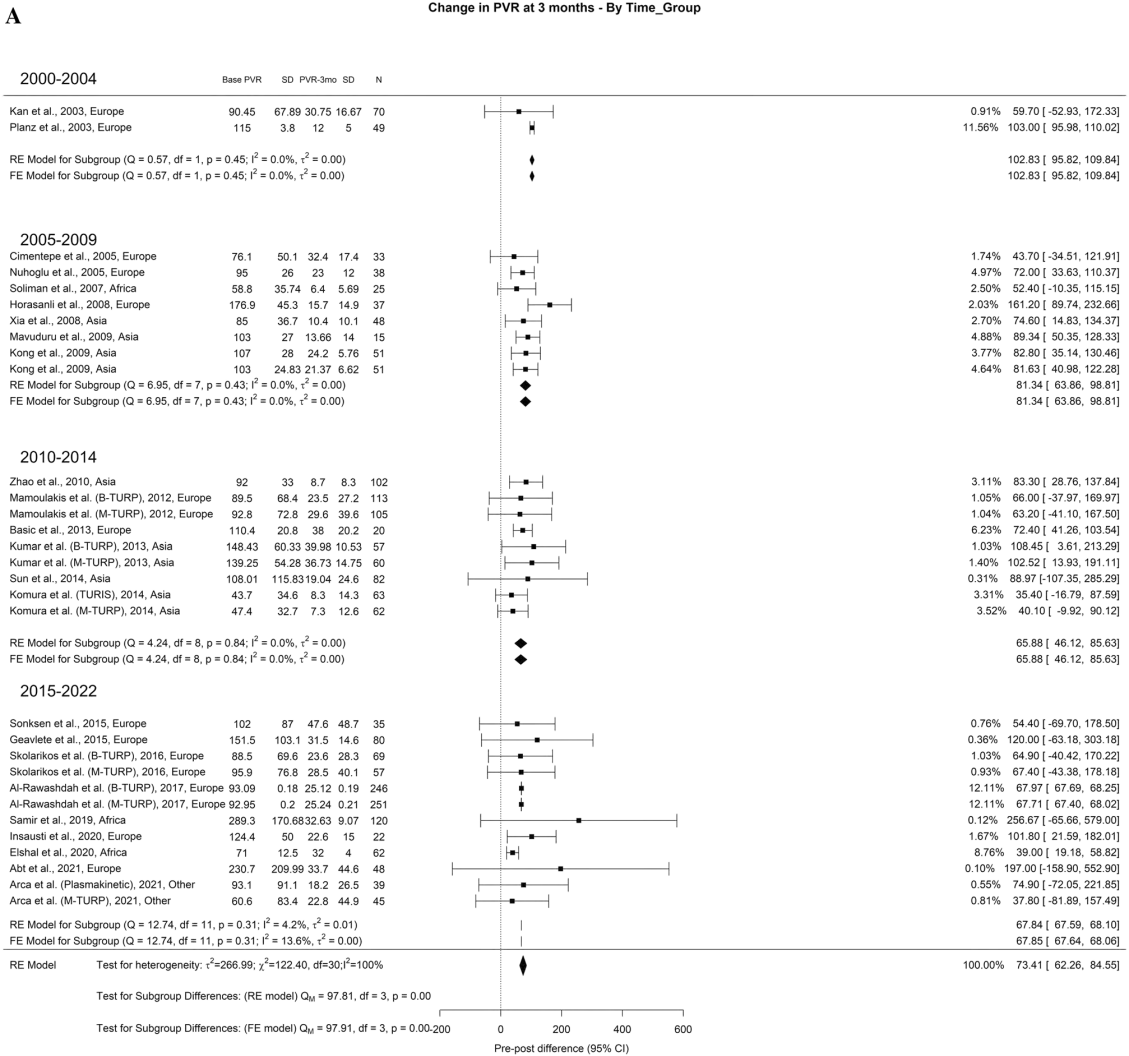

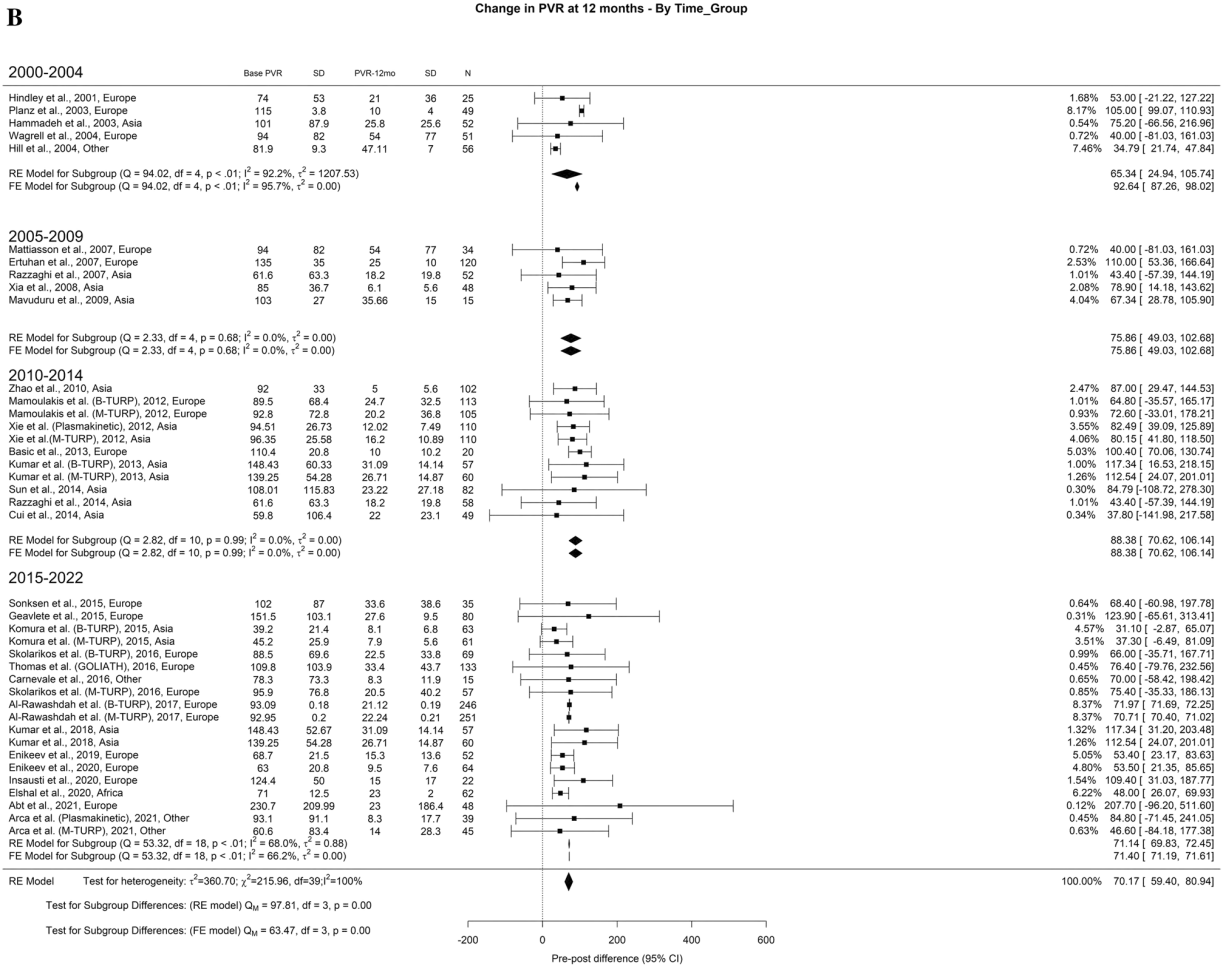

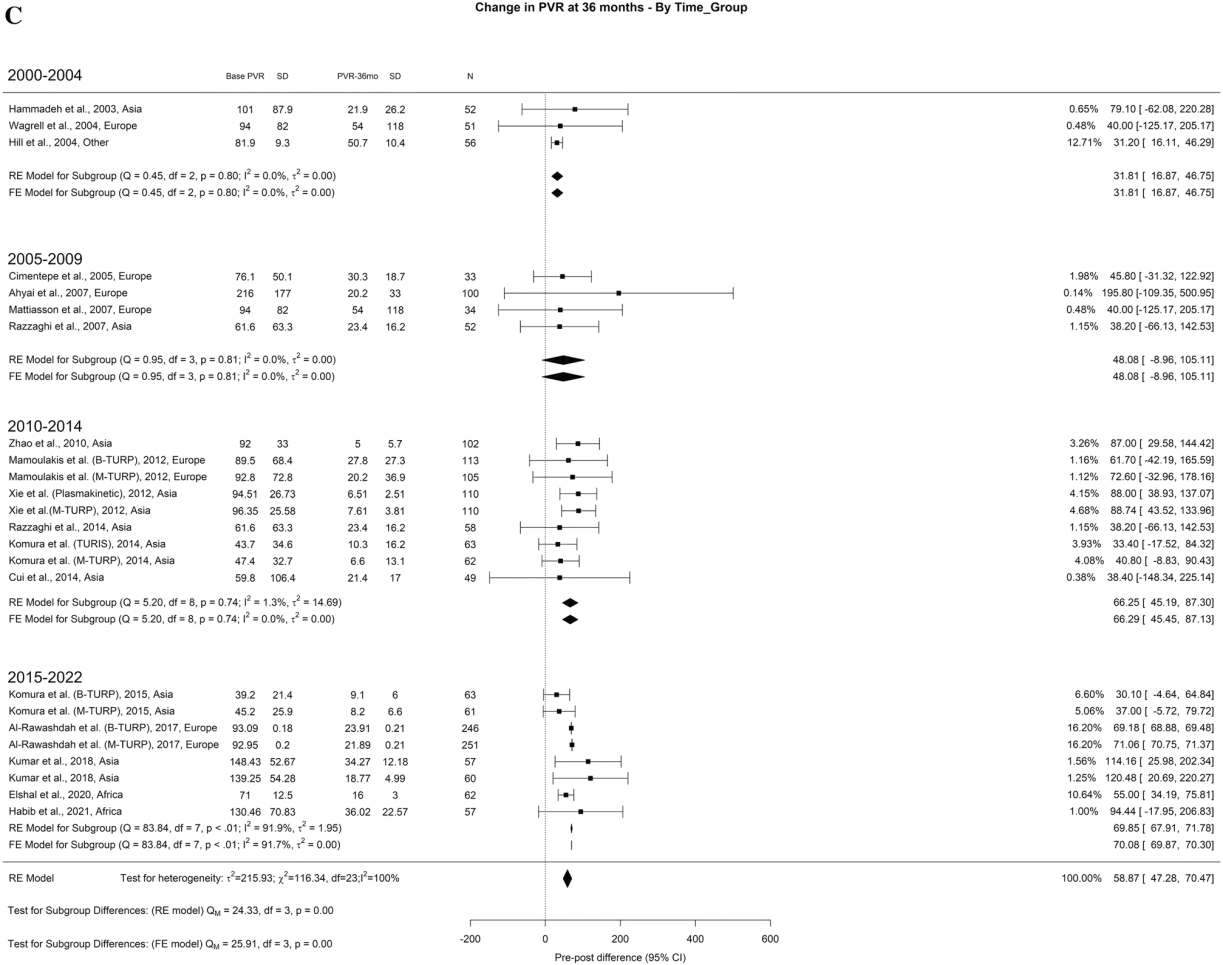
Change in the PVR by time group. A. At 3 months; B. At 12 months; C. At 36 months

When analyzing the PVR results, we found an overall decrease at 3-months, 1-year, and > 3-years of 71.73 ml, 72.94 ml, and 69.78 ml, respectively. The overall heterogeneity (OH) was elevated (I^2^ = 100%) in all perspectives of analysis. A difference between the groups was seen at all periods, in which we found significant differences in all follow-up periods. At 3-months (Qm = 97.91, p = 0.00), 2000–2004, 2005–2009, 2010–2014, 2015–2022 had 102.83, 81.34, 64.09, and 67.84. Looking at 1-year (Qm = 97.91, *p* = 0.00), 2000–2004, 2005–2009, 2010–2014, 2015–2022 had 64.34, 75.86, 88.88, and 71.14 ml, respectively. Finally, at 3-years (Qm = 43.81, *p* = 0.00), 2000–2004, 2005–2009, 2010–2014, 2015–2022 had 31.81, 48.08, 65.97, and 69.85 ml, respectively (Fig. 3A–C).

The better improvement in IPSS and Qmax at > 3-year in the later part of the study period might be explained by the gradual replacement of monopolar (M-TURP) with bipolar (B-TURP) over the last two decades. We noted that during 2000–2004, no studies utilized bipolar energy as compared to 12%, 46%, and 48% during 2005–2009, 2010–2014, and 2015–2022, respectively. Although previous systematic reviews and meta-analysis comparing M-TURP and B-TURP revealed no difference in the outcomes within 12 months [112–116], there is a lack of studies comparing long-term durability between these two technologies. Moreover, as noted in our studies, variability in assessing this variable can explain the high OH regarding PVR estimation. Despite the differences being statistically significant, the differences do not appear clinically significant [117].

### Sexual outcomes: SHIM, erectile dysfunction, and retrograde ejaculation

**Fig. 4:**
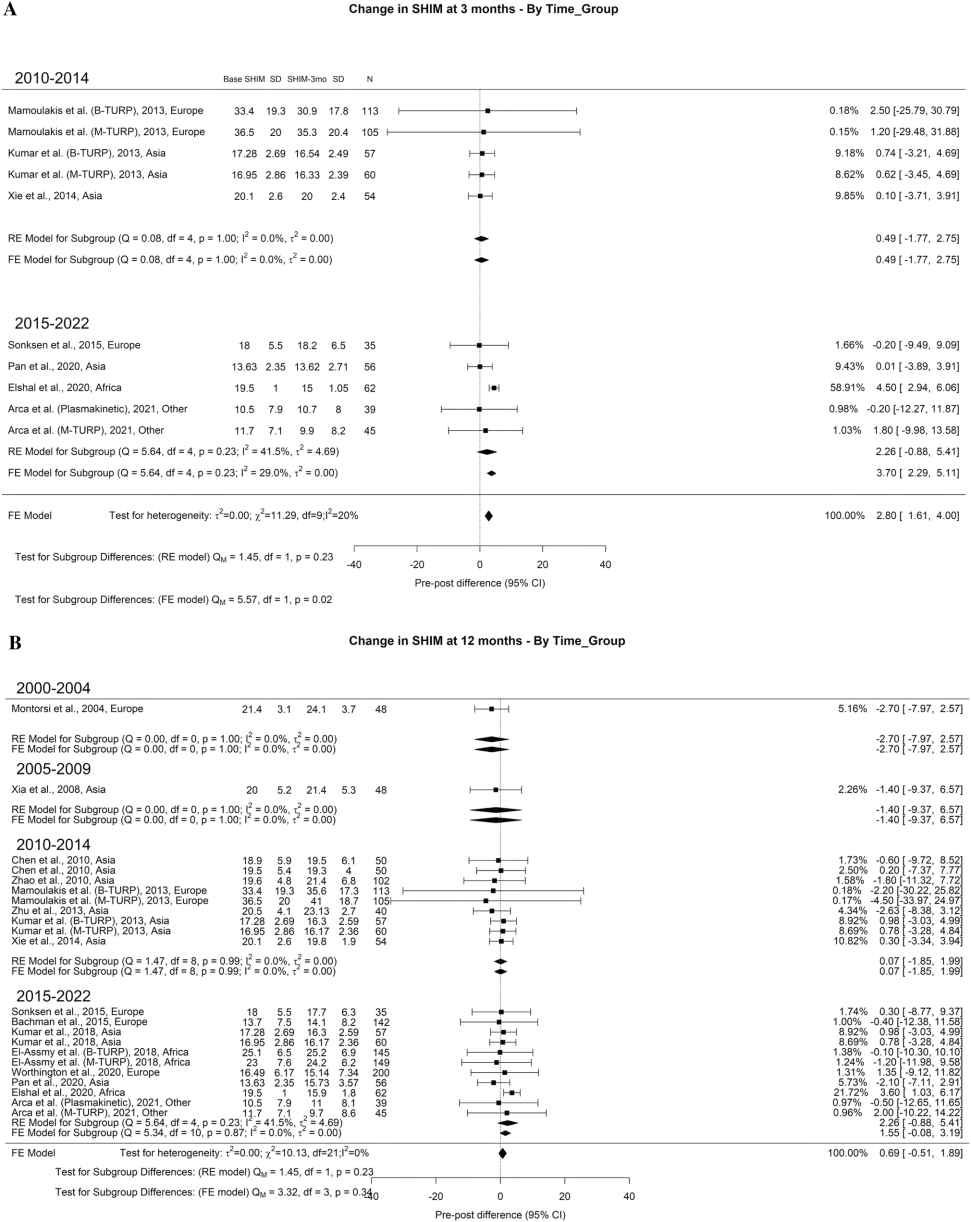

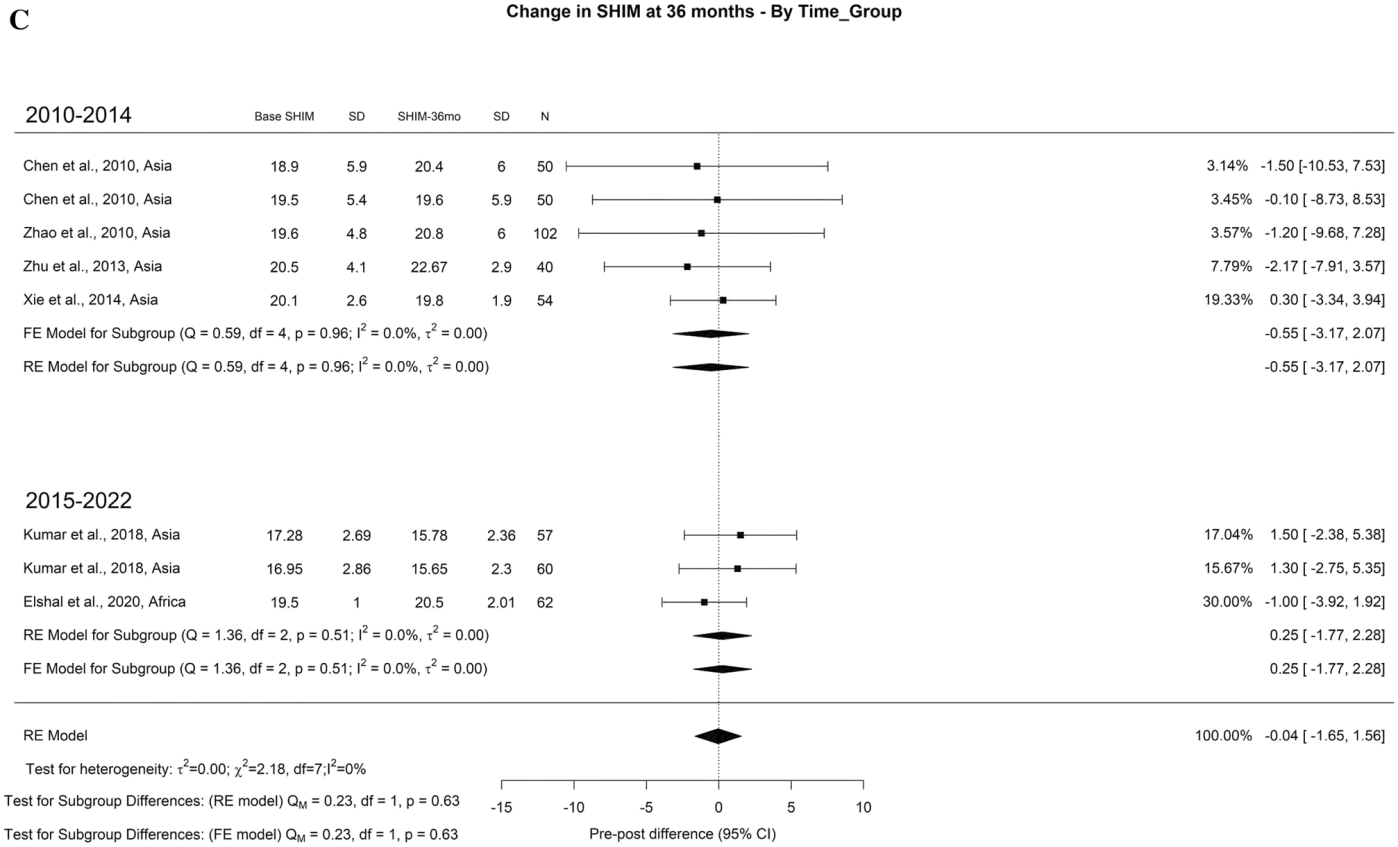
Change in the SHIM by time group. A. At 3 months; B. At 12 months; C. At 36 months

**Fig. 5:**
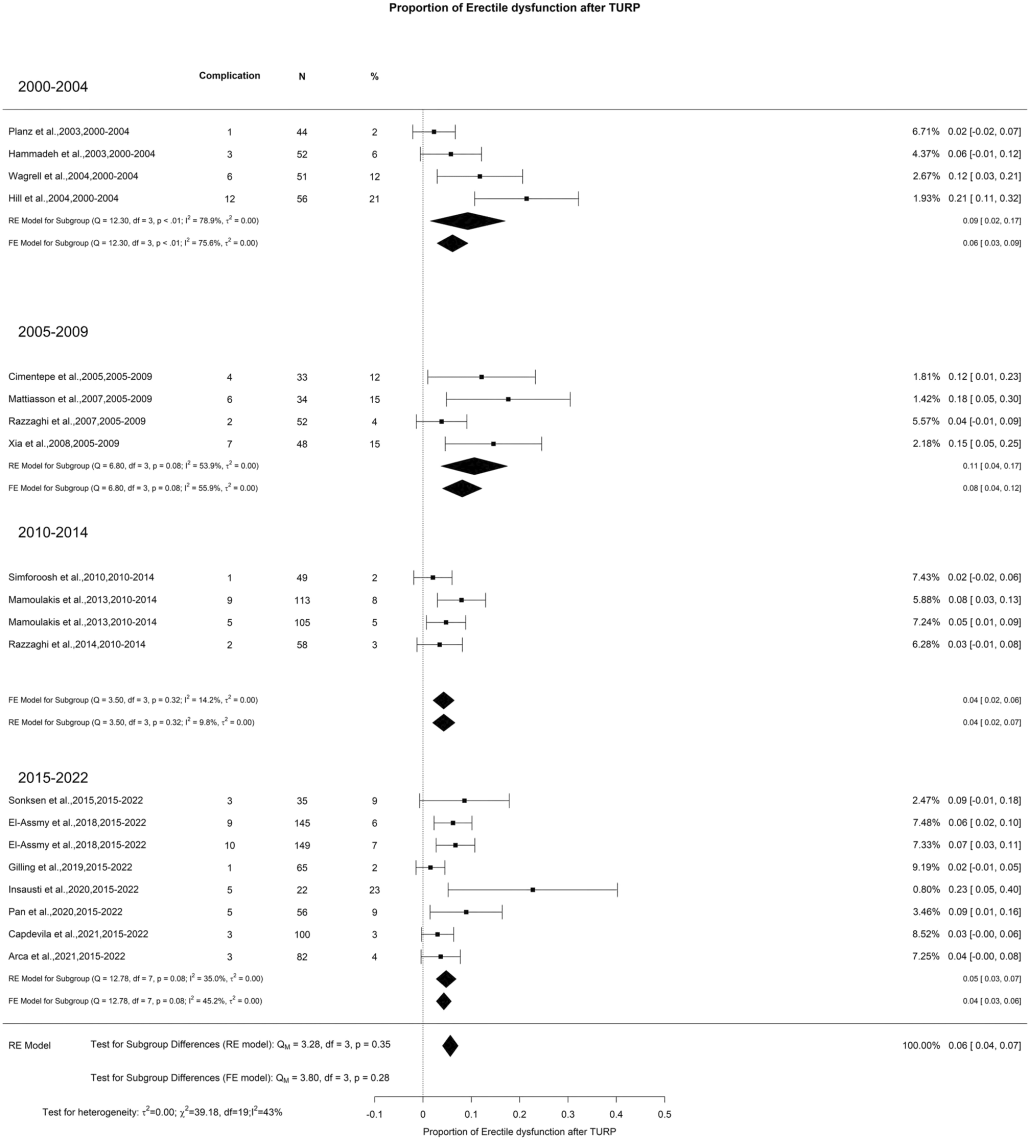
Proportion of Erectile Dysfunction after TURP

**Fig. 6:**
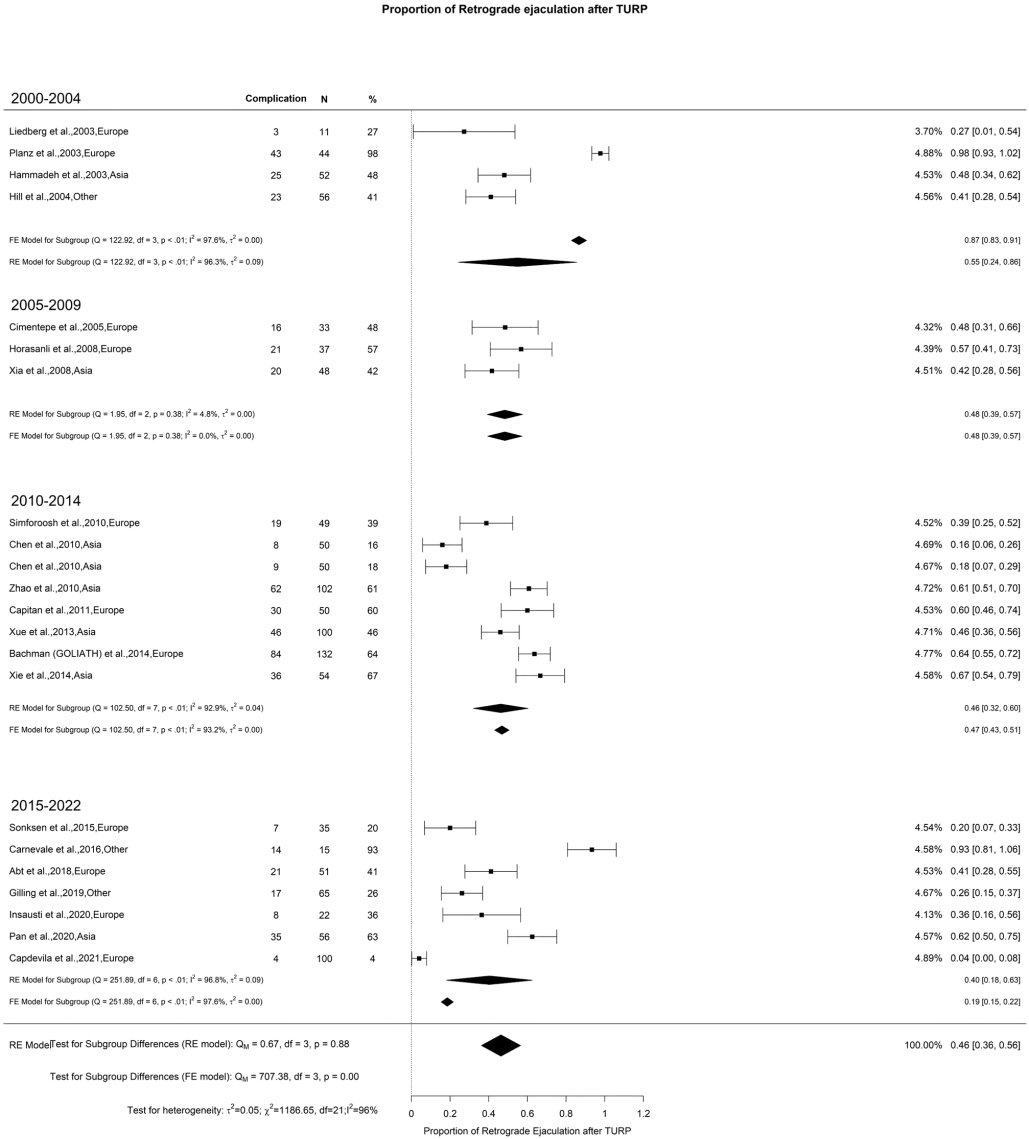
Proportion of Retrograde Ejaculation after TURP

There was an overall decrease in SHIM scores at 3-month and 1-year of 2.80 and 0.69 points, respectively. However, at > 3-years of follow-up, there was non-statistically significant overall increase in SHIM score of 0.4 points (Fig. 4A–C). The overall incidence of ED reported after TURP was 6% with moderate OH (I^2^ = 43%) (Fig. 5). Regarding retrograde ejaculation, the overall incidence reported was 46% with high OH (I^2^ = 96%) (Fig. 6). There was no significant difference in the SHIM score analysis or significant findings in the incidence of ED and retrograde ejaculation.

Our results align with several studies showing that TURP does not negatively impact erectile function and may lead to improvement [118–122]. The reported incidence of retrograde ejaculation after TURP is 50–70% [123]. Technical modifications that include preserving peri-montanal tissue to preserve antegrade ejaculation are described in the literature, but we did not find any RCT that analyzed these technical modifications during the study period [124, 125].

### Early postoperative complications—TURP syndrome

**Fig. 7:**
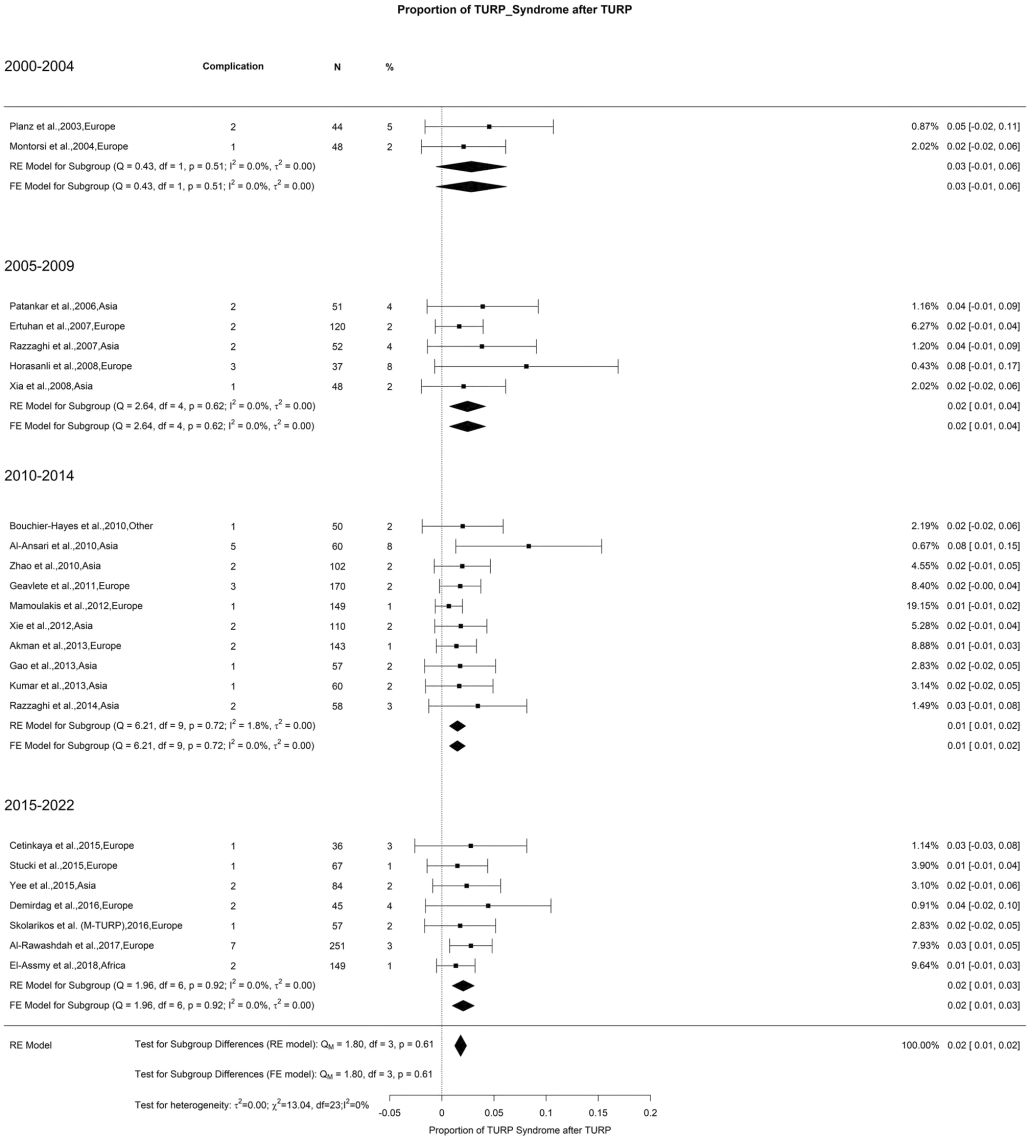
Proportion of TURP syndrome after TURP

The overall rate of TURP syndrome after prostate resection in the last 20 years was 2%. Other authors also noted that TURP syndrome has an incidence of 0.5–8%, with a declining trend [126]. The introduction of B-TURP with normal saline as irrigation fluid represents one of the main factors responsible for the decline in this severe and potentially life-threatening complication [127] (Fig. 7). However, in the present study we did not find statistically significant difference in the risk of this syndrome, which might be attributable to overall low incidence of TURP syndrome in all included studies.

### Early postoperative complications—bleeding, blood transfusion, and clot evacuation

**Fig. 8:**
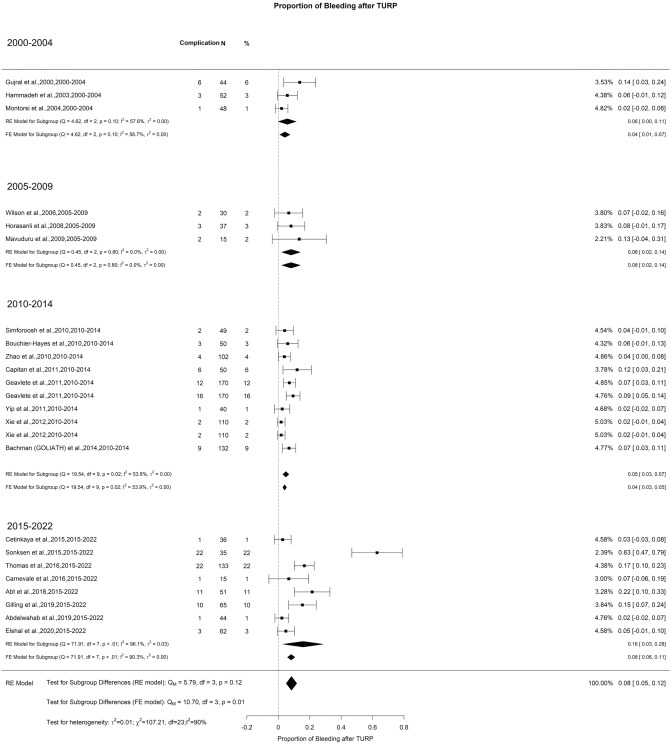
Proportion of Bleeding after TURP

**Fig. 9:**
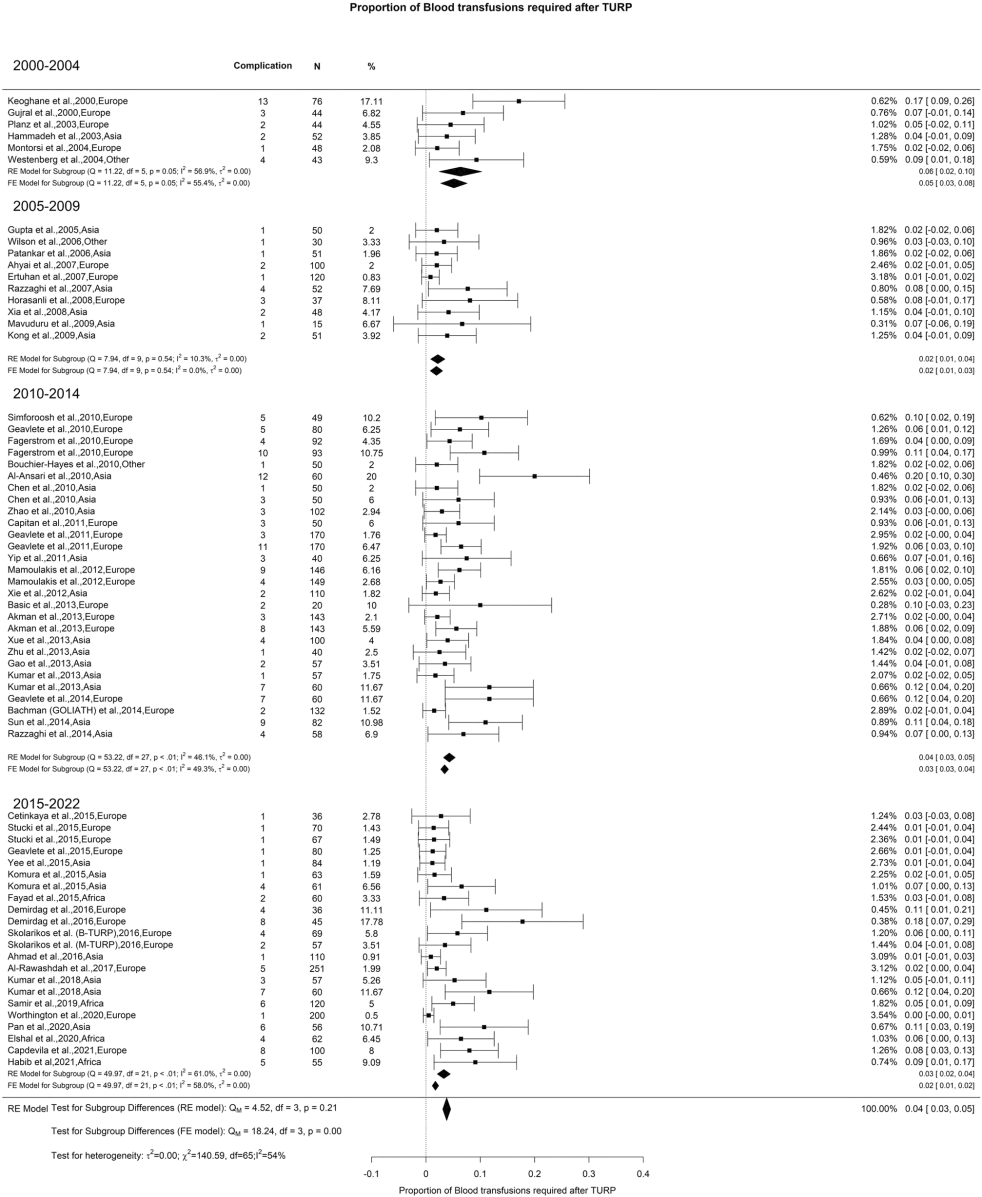
Proportion of Blood Transfusion after TURP

**Fig. 10:**
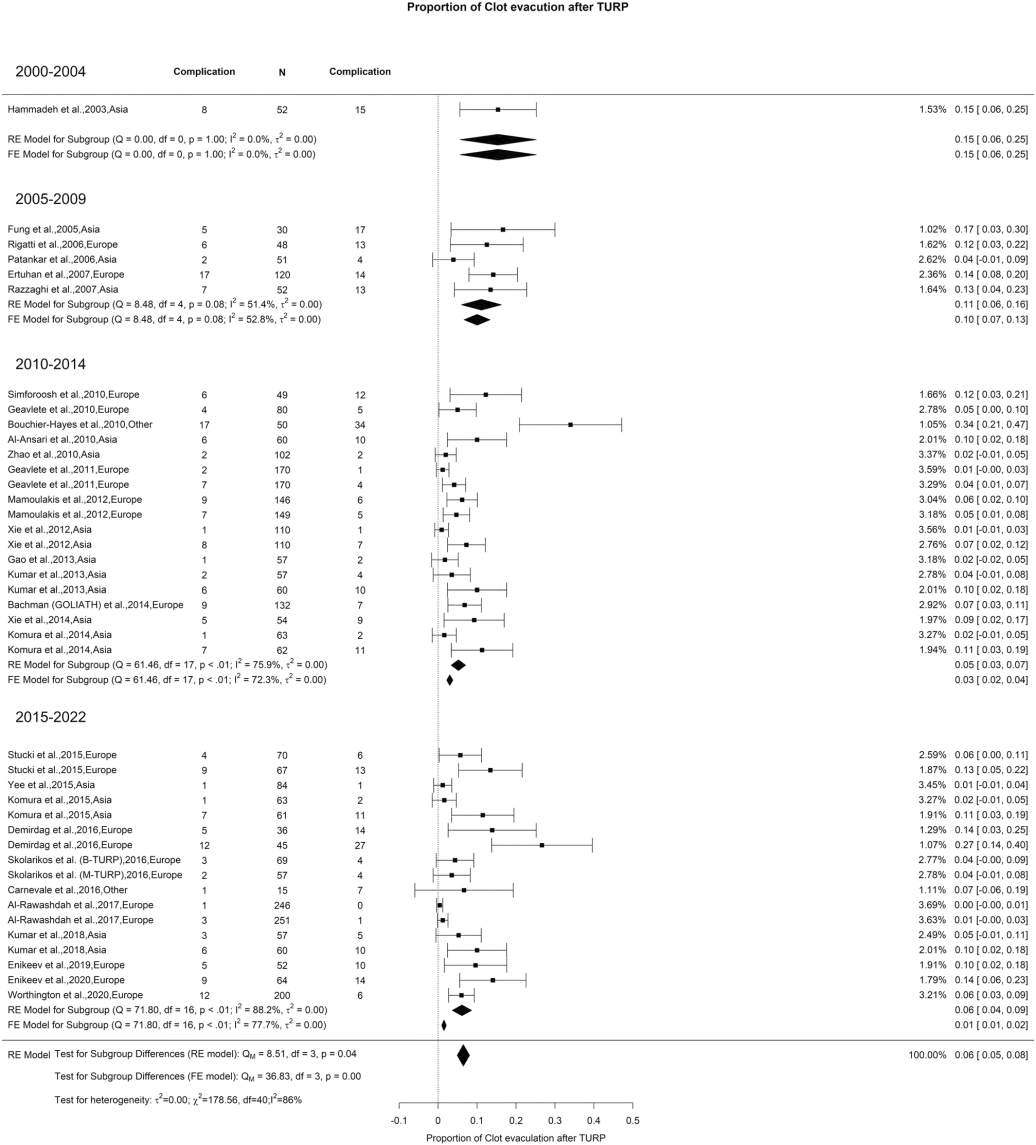
Proportion of Clot Evacuation after TURP

We noted an overall risk of bleeding reported after TURP of 8% with high OH (I^2^ = 93%). The reported incidence in 2000–2004, 2005–2009, 2010–2014, and 2015–2022 (Qm = 3.13, *p* = 0.37) was 6%, 8%, 5%, and 16%, respectively (Fig. 8). However, although the overall rate of blood transfusion as well as the need for clot evacuation after TURP was 6% with high OH (I^2^ = 86%, in both situations), we did not notice any difference in blood transfusion requirement (Qm = 1.43, *p* = 0.7) (Fig. 9). However, there was a significant difference in clot evacuation incidence. The reported rate was 15%, 11%, 5%, and 6% for the period of 2000–2004, 2005–2009, 2010–2014, and 2015–2022, respectively (Qm = 8.51, *p* = 0.04) (Fig. 10).

The main reason for the high OH in the reported incidence of bleeding was the variation in including intraoperative and/or postoperative hematuria while reporting the bleeding outcome. There was a lack of clear definition in the studies regarding objective and replicable quantification of bleeding. Since the indication for blood transfusion after TURP is usually standardized, no significant difference was noted in its incidence in this temporal analysis. We also noted a significant drop in the incidence of clot evacuation, possibly representing improved hemostasis with newer technical advances in TURP.

### Early postoperative complications: urinary retention

**Fig. 11:**
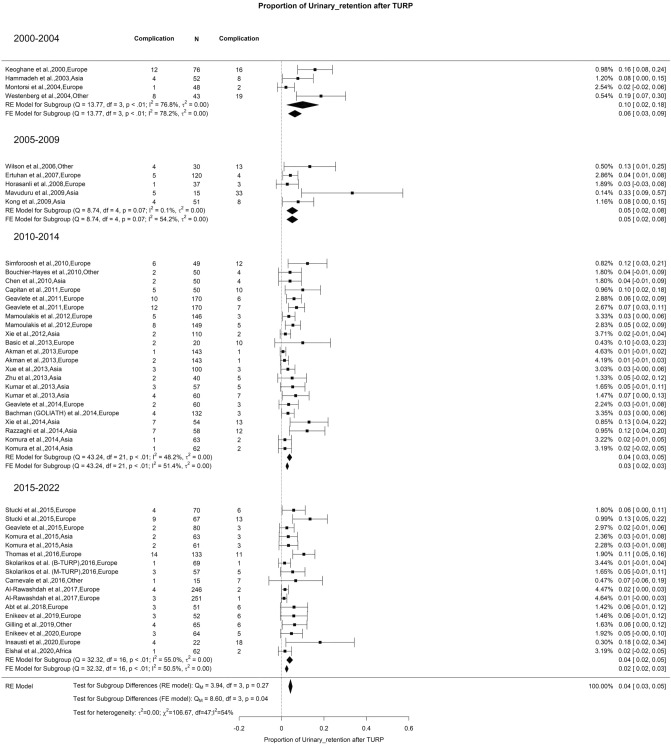
Proportion of Urinary Retention after TURP

The overall rate of urinary retention after TURP was 4% with moderate OH (I^2^ = 54%) (Fig. 11). There was no significant difference between the groups. The results were similar at 5.8% in 2008, as noted by Reich et al. in an extensive series of 10,654 patients. These authors noted that patients catheterized before surgery were at risk of the highest rate of urinary retention after TURP [128].

### Early postoperative complications: urinary tract infection

**Fig. 12:**
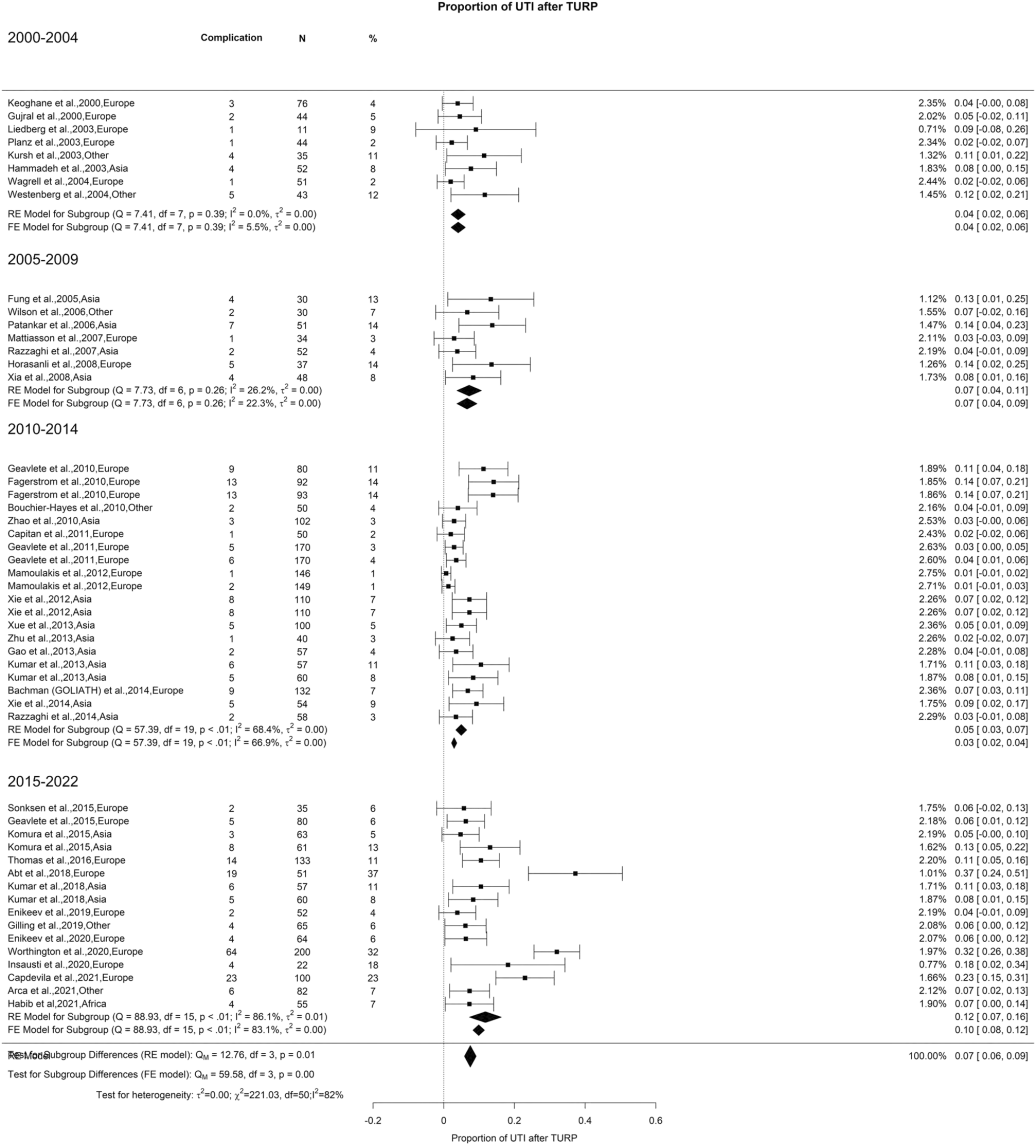
Proportion of Urinary Tract Infection after TURP

We noted an overall incidence of UTI of 8% with high OH (I^2^ = 81%). A significant difference was found (Qm = 12.04, *p* = 0.01), wherein 2000–2004, 2005–2009, 2010–2014, and 2015–2022 had 4%, 7%, 5%, and 12% of UTI, respectively (Fig. 12).

Prima facie, it appears that UTI rates are increasing. However, it is essential to note that there is a lack of consistent and clear definition of this complication in most studies, without clear information on whether the UTIs were symptomatic or asymptomatic, and whether the diagnosis relied solely on clinical symptoms or if it was confirmed through a positive urine culture. Postoperative UTI remains a significant concern for patients, constituting one of the most common reasons for hospital readmission within 30 days after TURP [129].

### Early postoperative complications: irritative symptoms

**Fig. 13:**
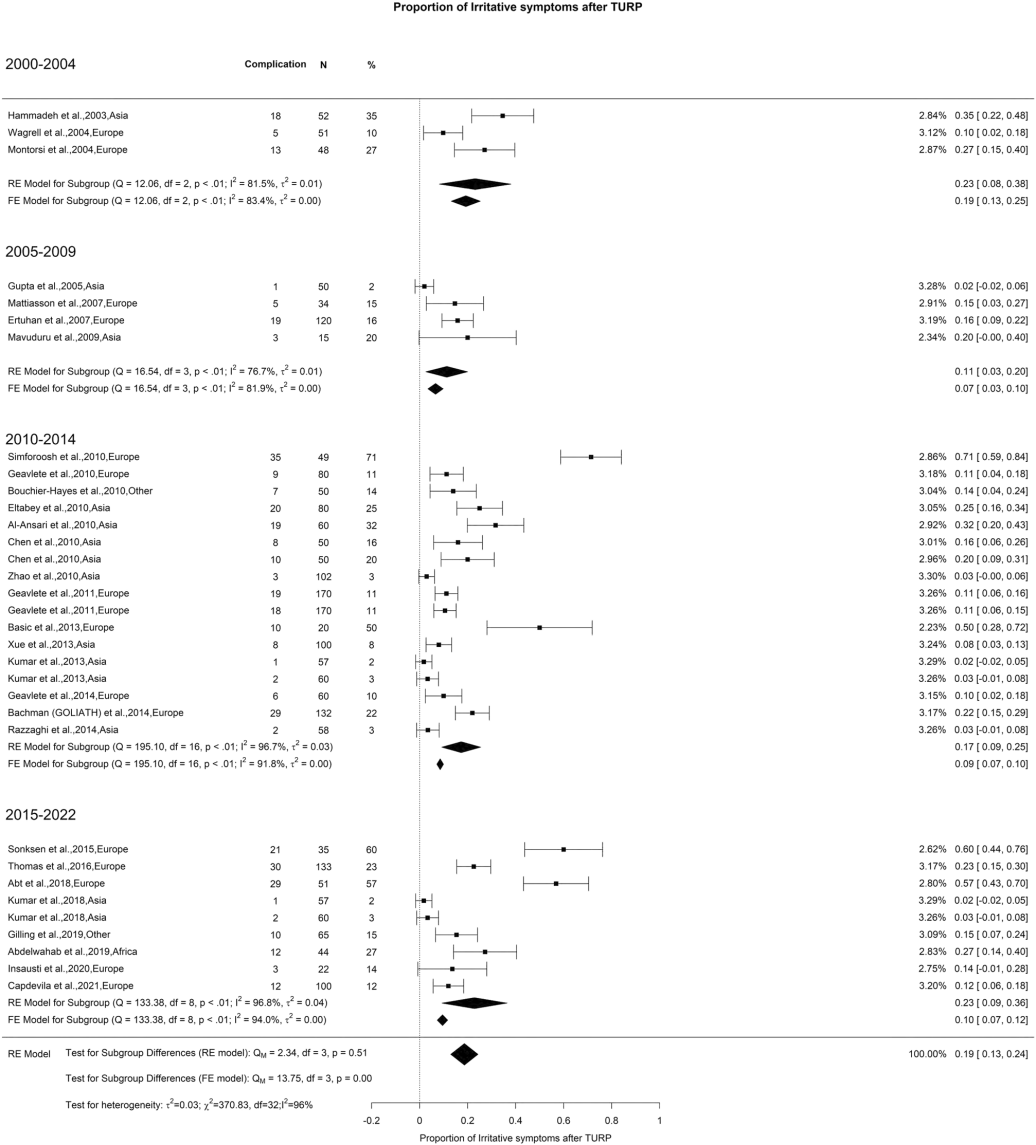
Proportion of Irritative Symptoms after TURP

We noted an overall rate of irritative symptoms after TURP of 19% with high OH (I^2^ = 96%) but there was no significant difference found in our analysis (Fig. 13).

### Early postoperative complications: urinary incontinence

**Fig. 14:**
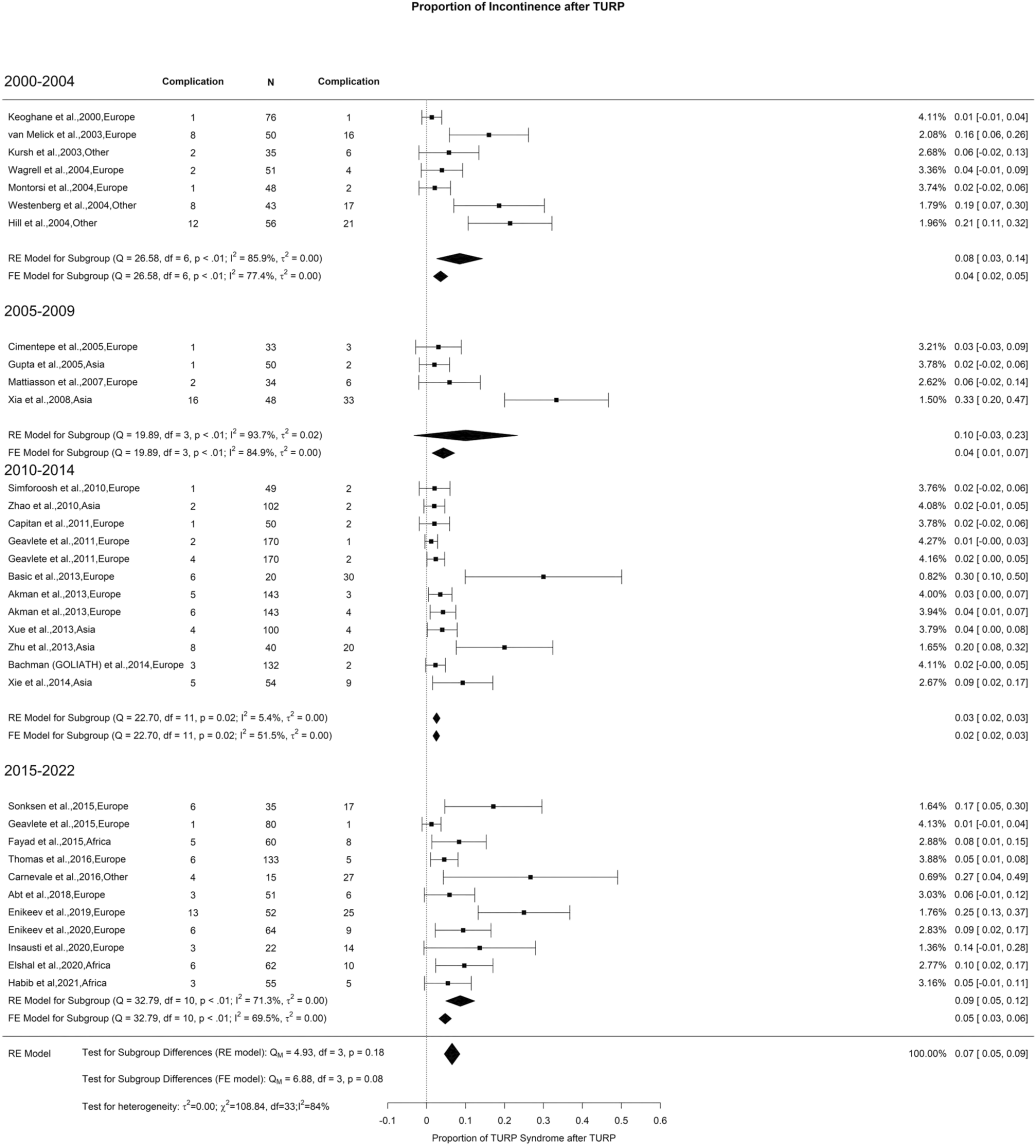
Proportion of Urinary Incontinence after TURP

The overall rate of urinary incontinence reported in all studies included was 8%, with high OH (I^2^ = 84%). We did not notice any significant change in the incidence of incontinence over the study period (Fig. 14).

In the present study, we did not conduct separate analyses of the subtypes of urinary incontinence or specify the postoperative follow-up period for reporting incontinence due to the considerable variability observed in different studies. However, it is crucial to acknowledge that incontinence following benign prostatic obstruction (BPO) procedures significantly impacts patients’ quality of life, with age being a significant risk factor [130, 131]. It is essential to establish a standardized reporting approach in all relevant studies to advance the understanding of postoperative incontinence in the context of various BPO surgeries. Although the literature suggests a comparable incontinence rate across all treatment modalities for BPO, there is a consistent trend of improvement in the postoperative period over time [132].

### Late postoperative complications: urethral stricture

**Fig. 15:**
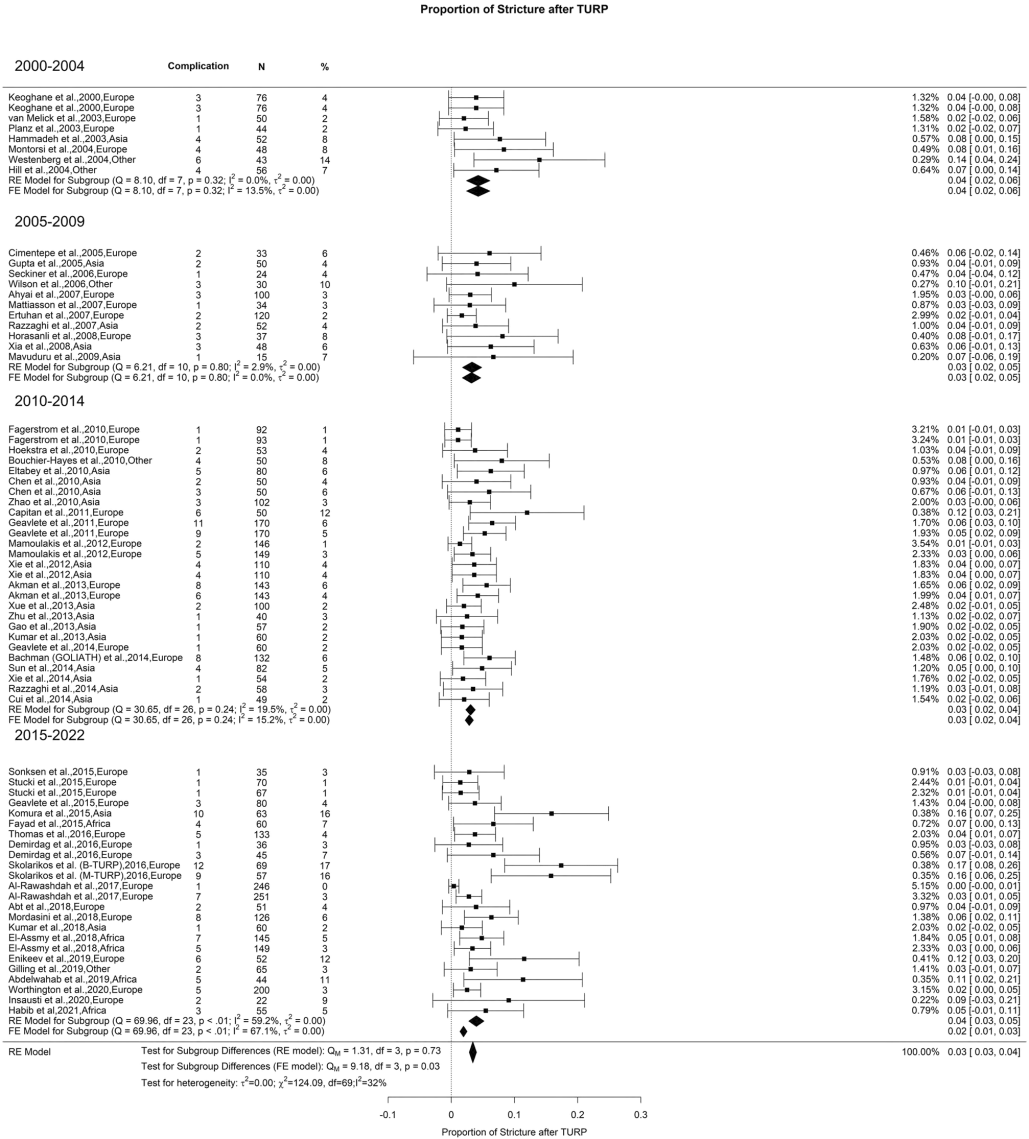
Proportion of Urethral Stricture after TURP

In the present study, the overall US rate after TURP was 3% with moderate OH (I^2^ = 32%) with no significant difference between the groups (Fig. 15). The reported incidence of US after TURP in the literature ranges from 2.2% to 9.8% [127], and according to a study conducted by Pirola et al., TURP has the highest incidence of US when compared to enucleation and ablation procedures [133]. Some factors associated with this condition are the use of bipolar energy, instrument caliber, and duration of postoperative catheterization. However, the mechanisms and risk factors for stricture formation remain an area of active debate. Some factors implicated include a slow resection rate, prolonged duration of surgery, large prostatic volume, temperature of the irrigation solution, and the size and frequency of transurethral instrument passage [134–138].

### Late postoperative complications: bladder neck stenosis

**Fig. 16:**
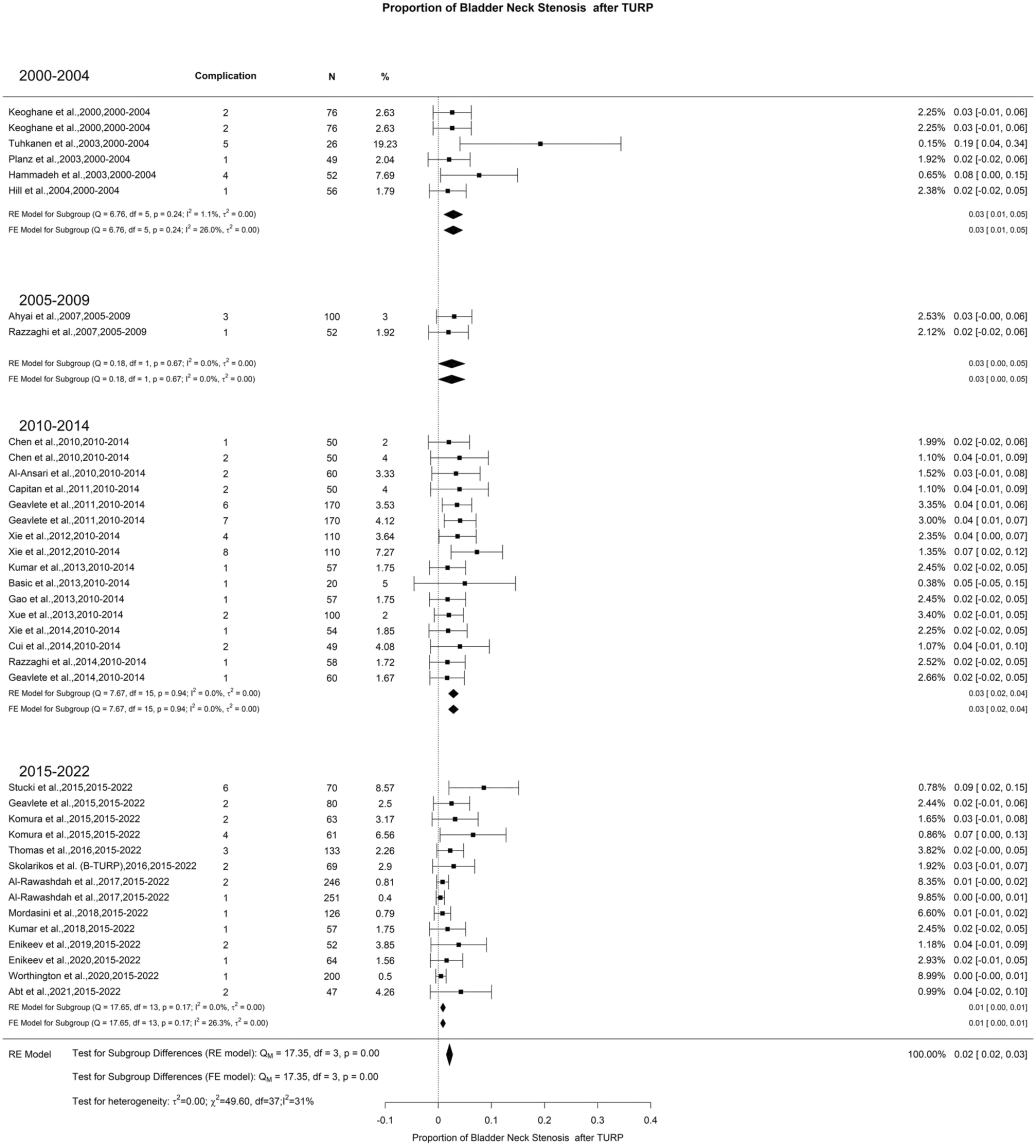
Proportion of Bladder Neck Stenosis after TURP

In our study, the overall BNS rate after TURP was 2% with moderate heterogeneity (I^2^ = 31%). A significant difference was found with 2000–2004, 2005–2009, and 2010–2014 showing BNS rate of 3% while, 2015–2022 demonstrated a rate of 1% (Fig. 16). It is well-documented in the literature that BNS is commonly associated with prostate sizes below 30 g, with reported incidence rates ranging from 0.3% to 9.2% [127, 139, 140]. Despite the similarity in the incidence of BNS after TURP, enucleation, and ablative techniques, it is important to note that the outcomes of endoscopic management to treat BNS can differ significantly depending on the primary procedure that caused this complication. BNS treatment success seems to be higher after endoscopic enucleation procedures (EEP) compared to TURP [141, 142].

### Incidental prostate cancer

**Fig. 17:**
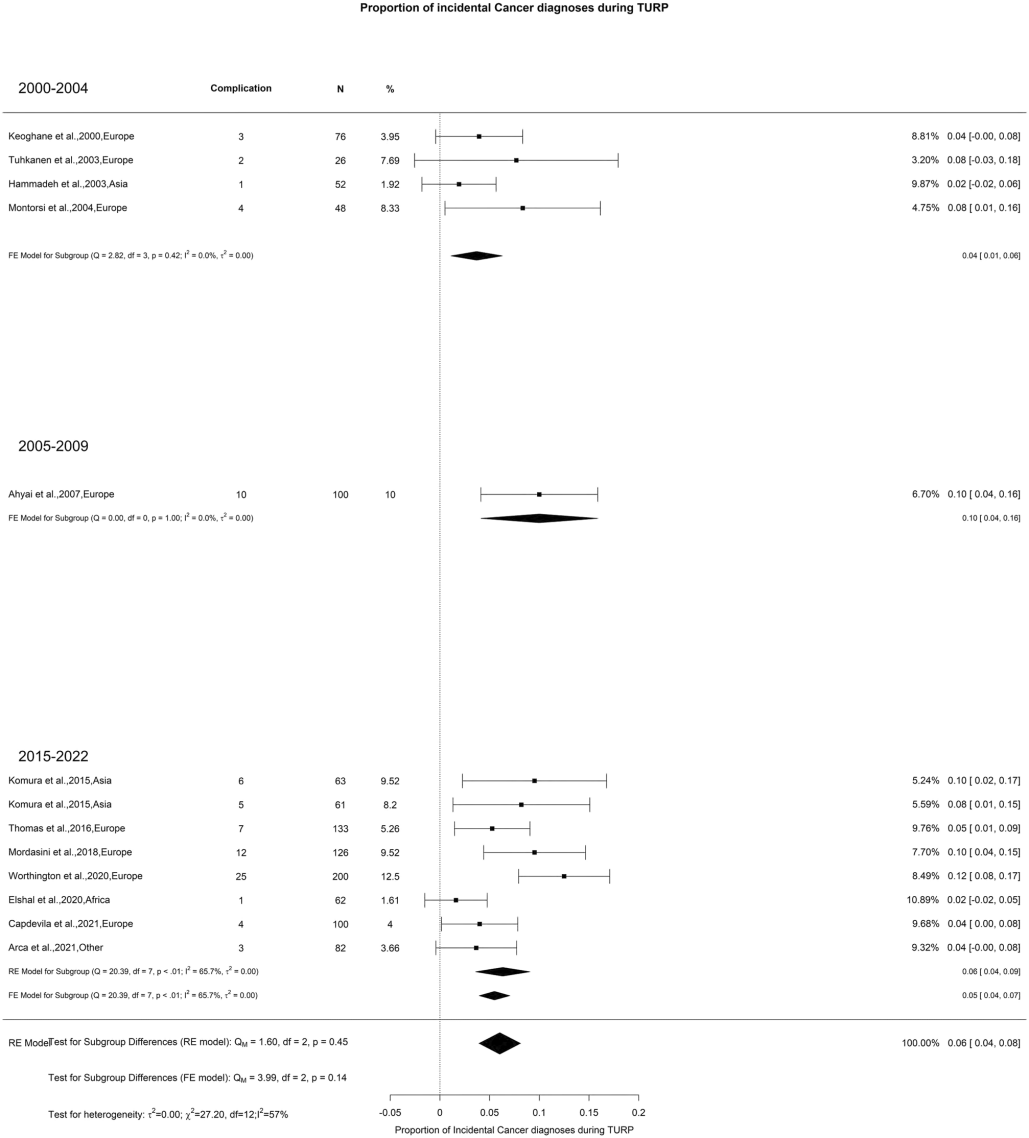
Proportion of Incidental Prostate Cancer after TURP

The overall diagnosis of iPCa after TURP was 6%, with moderate OH (I^2^ = 61%) and no difference seen in the distinct groups of analysis (Fig. 17). However, as noted by other authors, the incidence of iPCa is inferior with TURP than with EEP [143]. This might be attributed to the better prostate debulking achieved by EEP compared to TURP.

### Reduction in prostate volume, PSA, and retreatment rate

**Fig. 18:**
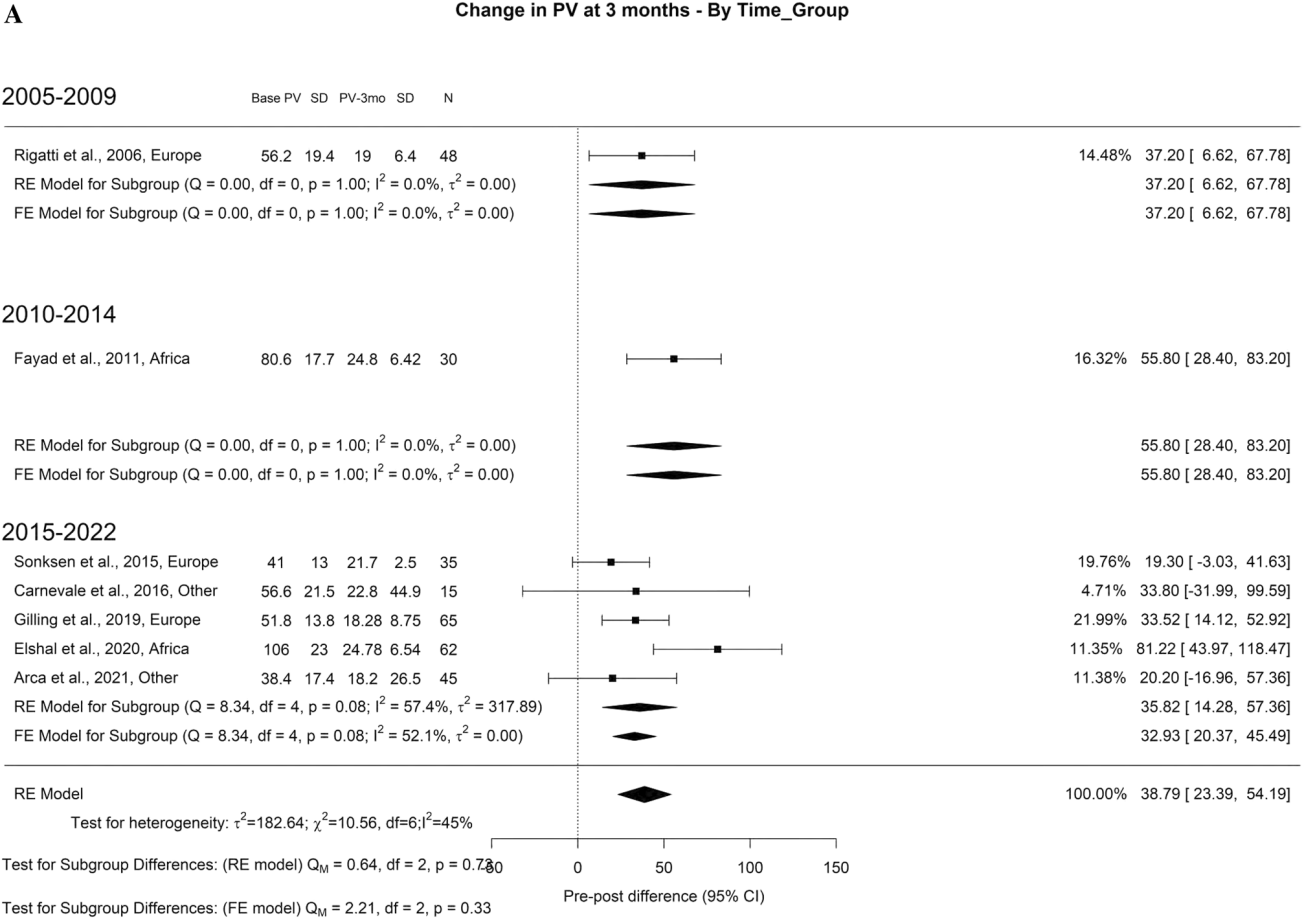

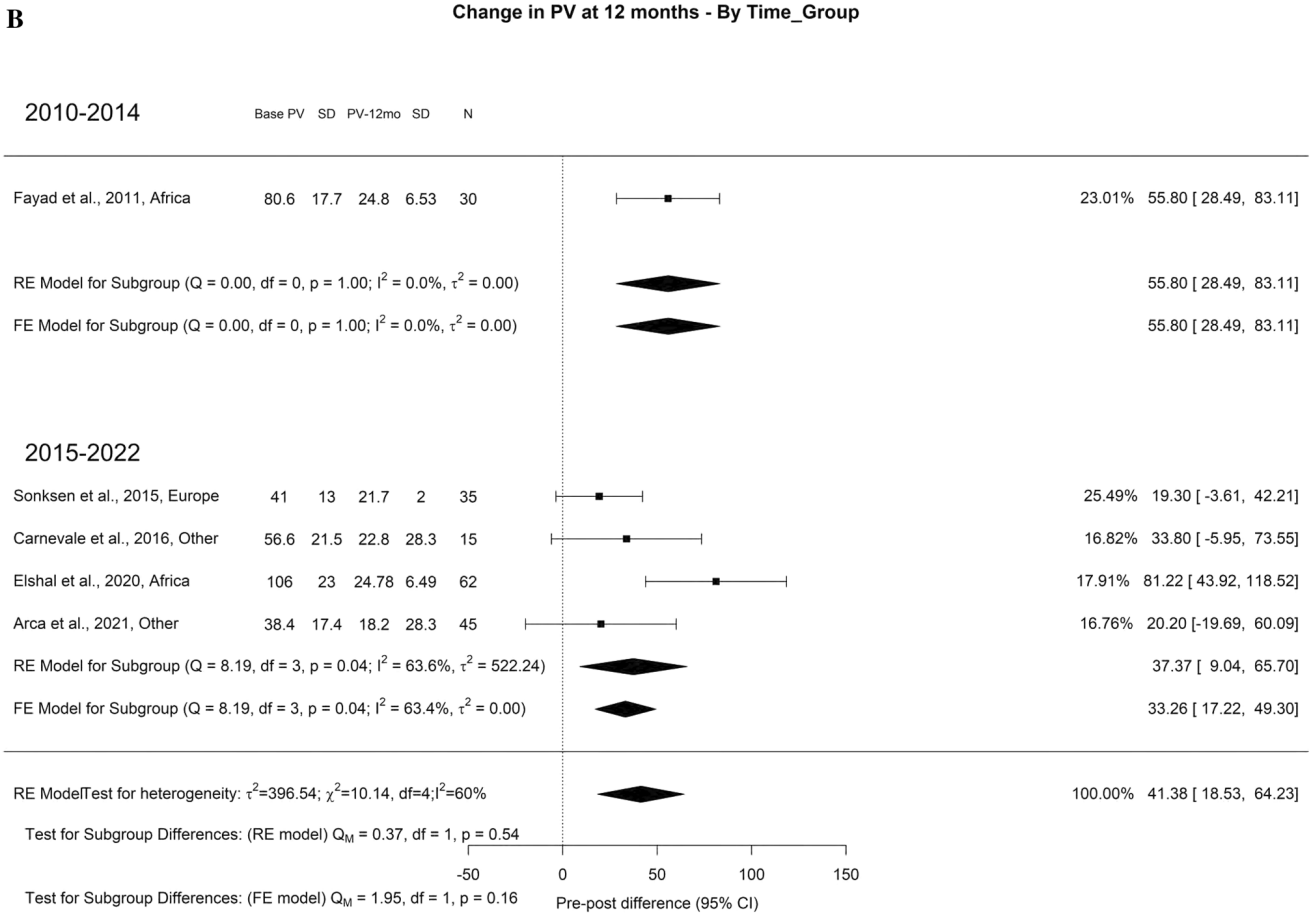
Change in the Prostate Volume by time group. A. At 3 months; B. At 12 months

The volume of residual prostate at 3-month and 1-year was 38.79 and 41.38 g, respectively. There was no significant difference in the heterogeneity of prostate volume reduction between the groups (Fig. 18A–B).

**Fig. 19:**
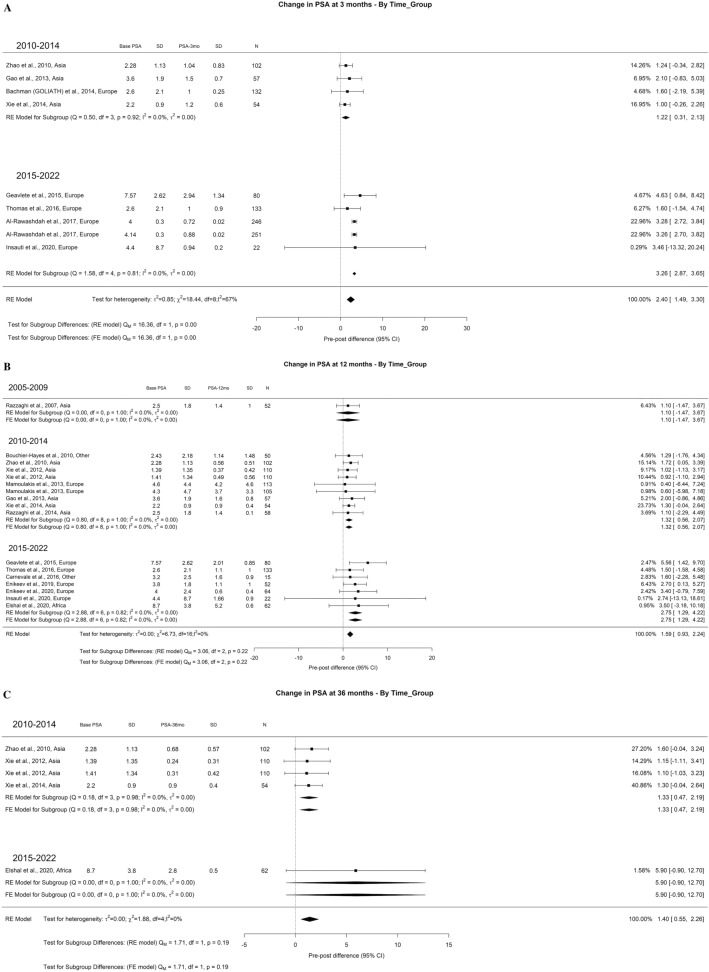
Change in the PSA by time group. A. At 3 months; B. At 12 months; C. At 36 months

Regarding PSA, we used the absolute values as it reflects completeness of resection and also serves as a baseline for prostate cancer screening for patients with iPCa. The overall decrease at 3-month, 1-year, and 3-year was 2.40, 1.59, and 1.40 ng/ml, respectively. At 3-months it had a high OH (I^2^ = 62%) and significant difference between the timeframes (Qm = 16.36, *p* = 0.00, I^2^ = 67%) with PSA difference of 1.22 and 3.26 ng/ml in 2010–2014 and 2015–2022, respectively. At 36 months (Qm = 1.75, *p* = 0.19), the PSA difference found was 1.33 and 5.90 ng/ml in 2010–2014 and 2015–2022, respectively (Fig. 19A–C).

**Fig. 20:**
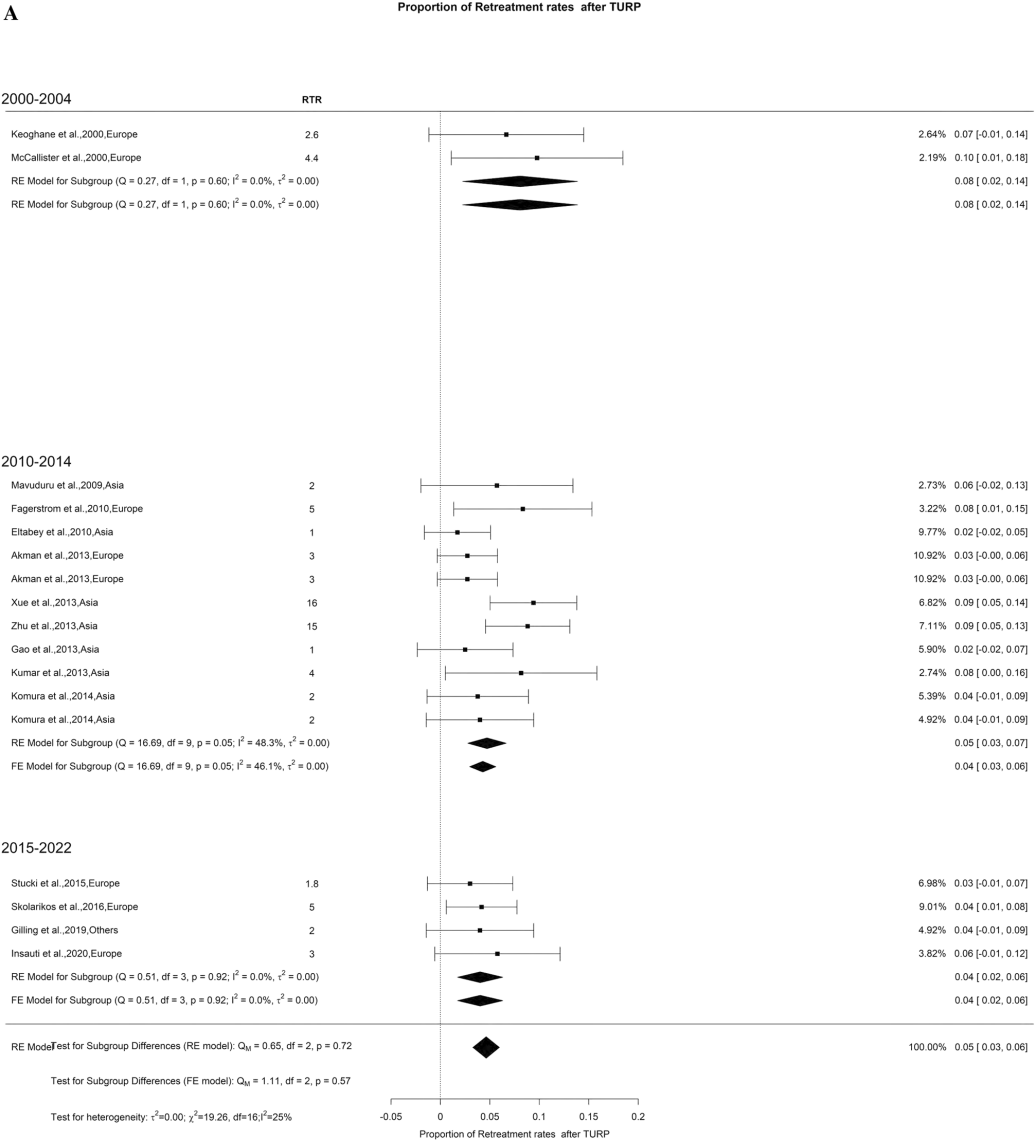

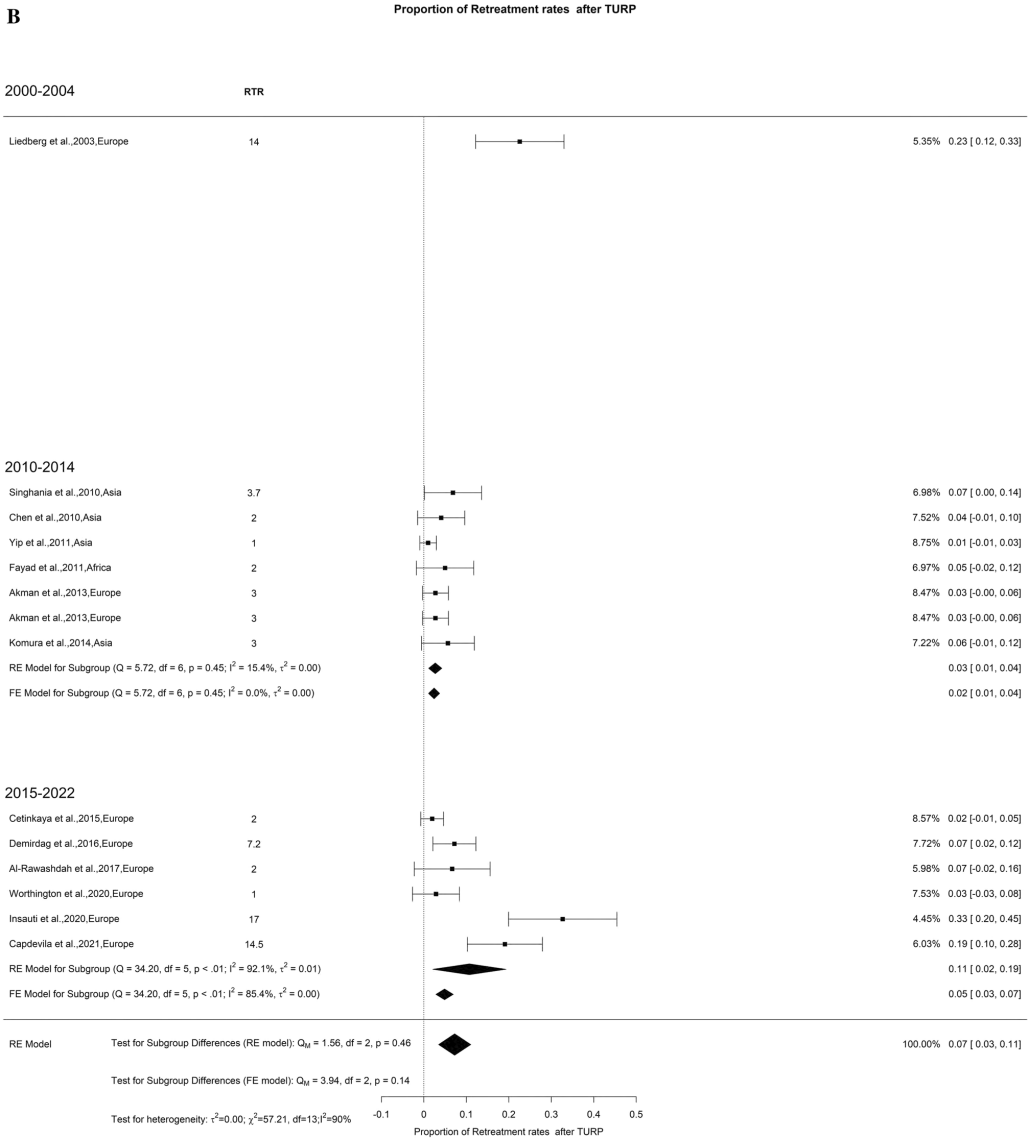
Retreatment Rate after TURP. A. At 12 months; B. At 36 months

We also assessed retreatment rates at 1 and 3 years, revealing a rate of 5% with low OH (I^2^ = 25%) and 7% with high OH (I^2^ = 90%), respectively. We did not find significant difference between the groups (Fig. 20A–B).

There is a historical lack of a precise definition regarding the optimal amount of tissue to be resected during TURP. This absence of a standardized template for assessing the completeness of surgery may result in significant variation in the final volume of resected tissue [144, 145]. As demonstrated by our results, the prostate gland will present a slow growth over time, and some factors such as age, testosterone, and inflammation markers play a crucial role [146]. Moreover, the impact of prostate volume on PSA is well described in the literature, and a general rule indicates a decrease of 0.1–0.3 ng/ml in PSA for each gram of tissue resected [145, 147–150]. As demonstrated by Bhat et al., various factors are related to the drop in the PSA after TURP, such as surgeon experience, prostate size, age, and patient comorbidities—from which prostate volume resected seems to be the most critical factor [145]. Similar trends related to prostate volume and PSA were seen in this study.

In a study by Frendl et al., treatment failure after TURP was defined as cases in which patients experienced acute urinary retention or required retreatment for lower urinary tract symptoms. The study found a 15.27% failure rate over seven years [151]. Similarly, in the study with the most extended follow-up to assess retreatment rates, Eredics et al. demonstrated that after eight years, the retreatment rate for TURP was 12.7% for cases performed between 2002 and 2006 [152]. When compared to a population that underwent TURP in the 90 s, the authors did not find a significant difference in the retreatment rate.

## Strengths and limitations

Our study presents several limitations. Firstly, it only includes RCTs published in the PubMed database. However, given the large number of RCTs (103 studies) we decided to restrict our search to one domain. We decided to include only RCTs to reduce heterogeneity of analysis to minimize bias and provide stronger evidence. Despite this, the heterogeneity of baseline population characteristics and values across the included studies may limit the generalizability of our findings. Additionally, the short-term follow-up in most studies may not provide a complete and comprehensive picture of TURP outcomes over time. Furthermore, variations in surgical techniques and post-operative care protocols among the different studies could introduce bias into our analysis. Another limitation stems from the differences in inclusion criteria used in clinical trials, with approximately 40% excluding patients without a clearly defined concept, leading to disparities in measuring treatment efficacy, such as retreatment rates [117, 153].

Furthermore, our study did not differentiate between TURP subtypes, such as monopolar, bipolar, and plasmakinetic, which might influence TURP’s effectiveness and the incidence of complications. For instance, different modalities have varying rates of complications, such as TURP syndrome and hemorrhage. Nonetheless, our goal was to discuss TURP in general, as there is no evidence of significant differences in efficacy between subtypes. Comparing surgical interventions in M-TURP or B-TURP series regarding efficacy measures is a reasonable approach.

Moreover, it is important to note that most RCTs were conducted in large tertiary care centers, potentially limiting the representation of TURP’s broader community practice, techniques, efficacy, and safety. Also, given the significant differences in hospitalization length and postoperative catheterization protocols across countries and hospitals, we excluded this variable from our analysis. Despite these limitations, our study offers valuable insights into variations in TURP outcomes and underscores the need for further research to address these limitations and enhance patient care. For instance, the higher heterogeneity observed in bleeding, clot evacuation, incontinence, irritative symptoms, and UTI emphasizes the need for more standardized reporting of outcomes in clinical trials evaluating TURP. As for the strengths of our study, it constitutes a comprehensive analysis of multiple studies, providing a broader view of TURP outcomes. Furthermore, our study contributes to the existing knowledge base by highlighting the variations and disparities in reported outcomes, laying the groundwork for future research aimed at improving the overall efficacy and safety of TURP.

## Future perspectives for research

The present meta-analysis highlights significant heterogeneity in outcomes reporting across studies, particularly concerning complications such as bleeding and irritative symptoms. To address this issue, future clinical trials should adopt standardized outcome measures and reporting criteria, to enhance the comparability of studies and enable more robust analyses. Establishing a consensus set of core outcomes would significantly enhance the quality and reliability of future research in this field, ensuring more accurate and meaningful comparisons between studies.

With the rapid advancement of technology, new surgical techniques are emerging as potential alternatives. However, the final test for most new treatments is their performance compared to TURP, as the gold standard of BPO treatment. This meta-analysis reveals non-uniform reporting results across TURP studies, especially in adverse events, which decreases confidence in research findings about newer techniques, as the standard for reference is not being reported in a consistent and standardized fashion. This is of particular significance in light of the emergence of MISTs, which claim similar efficacy and fewer adverse events compared to TURP. Therefore, conducting comparative studies that evaluate the outcomes of these emerging interventions against TURP will be instrumental in identifying the most effective and least invasive treatment options.

Additionally, BPO has a significant impact on patients’ quality of life and healthcare costs. Future studies should incorporate assessments of patients’ quality of life, conduct cost-effectiveness analyses. By doing so, a comprehensive evaluation of these new interventions can be performed considering those parameters, which will contribute to informed decision-making and the optimal allocation of healthcare resources. We also noted very limited studies in the first decade provided long-term data (> 3 years RTR) as compared to second decade of study period. Additionally, only three studies provided outcomes more than 5 years (Fig. 20B). It is important to reinforce that more studies are needed with long term follow-up to evaluate durability of procedure.

## Conclusions

This study demonstrates that in the last 20 years, the overall outcome of TURP remained unchanged. We highlighted that self-reported outcome measures were relatively consistent, while objective metrics varied significantly, with PVR and Qmax showing the most significant variation. The heterogeneity may indicate a lack of standardization in TURP. However, symptom improvement among patients is relatively uniform, which may explain why TURP is a historical benchmark procedure for BPO.

## Supplementary Information

Below is the link to the electronic supplementary material.

Supplementary file1 (DOCX 46 KB)

Supplementary file2 (DOCX 16 KB)

## Data Availability

No datasets were generated or analysed during the current study.
