# Supplementary material for: Evaluating transurethral resection of the prostate over twenty years: a systematic review and meta-analysis of randomized clinical trials
Source: World J Urol. 2024 Nov 15;42(1):639. doi: 10.1007/s00345-024-05332-3 (PMC11568034; doi:10.1007/s00345-024-05332-3)
Supplement: Supplementary file 1 — Supplementary file1 (DOCX 46 KB) [file 345_2024_5332_MOESM1_ESM.docx]

Supplementary Figure 1. PRISMA 2020 flow diagram for systematic reviews

**Identification of studies via databases and registers**

Records (from January 1^st^, 2000, to December 31^st^, 2022) identified through database searching **PubMed** (n = 551)

**Identification**

Records screened

(n = 551)

Records excluded based on title.

(n = 248)

**Screening**

Records screened

(n = 303)

Records excluded based on abstract (n = 159)

**Eligibility**

Full-text articles excluded

(n = 41)

Full-text articles assessed for eligibility

(n = 144)

Studies included in qualitative synthesis.

(n = 103)

**Included**

Supplementary figure 1: Study selection process.
