# Supplementary material for: Evaluating transurethral resection of the prostate over twenty years: a systematic review and meta-analysis of randomized clinical trials
Source: World J Urol. 2024 Nov 15;42(1):639. doi: 10.1007/s00345-024-05332-3 (PMC11568034; doi:10.1007/s00345-024-05332-3)
Supplement: Supplementary file 2 — Supplementary file2 (DOCX 16 KB) [file 345_2024_5332_MOESM2_ESM.docx]

**Supplementary Table 1**. Comparison of TURP and Non-TURP Procedures Over Time

TURP: Transurethral Resection of the Prostate.

| **Period** | **Nº of Cases** | **Comparison TURP** | **Comparison Non-TURP** |
| --- | --- | --- | --- |
| 2000-2004 | 17 | 1 | 16 |
| 2005-2009 | 18 | 6 | 12 |
| 2010-2014 | 48 | 22 | 26 |
| 2015-2022 | 41 | 18 | 23 |
